# Data‐driven analysis of *JAK2*V617F kinetics during interferon‐alpha2 treatment of patients with polycythemia vera and related neoplasms

**DOI:** 10.1002/cam4.2741

**Published:** 2020-01-28

**Authors:** Rasmus K. Pedersen, Morten Andersen, Trine A. Knudsen, Zamra Sajid, Johanne Gudmand‐Hoeyer, Marc J. B. Dam, Vibe Skov, Lasse Kjær, Christina Ellervik, Thomas S. Larsen, Dennis Hansen, Niels Pallisgaard, Hans C. Hasselbalch, Johnny T. Ottesen

**Affiliations:** ^1^ Department of Science and Environment Roskilde University Roskilde Denmark; ^2^ Department of Haematology Zealand University Hospital Roskilde Denmark; ^3^ Department of Production, Research, and Innovation Region Zealand Sorø Denmark; ^4^ Faculty of Health and Medical Sciences University of Copenhagen Copenhagen Denmark; ^5^ Department of Pathology Harvard Medical School Boston FL USA; ^6^ Department of Laboratory Medicine Boston Children's Hospital Boston FL USA; ^7^ Department of Hematology Odense University Hospital Odense Denmark; ^8^ Department of Surgical Pathology Zealand University Hospital Roskilde Denmark

**Keywords:** early treatment, essential thrombocythemia, interferon‐alpha2, *JAK2*V617F kinetics, myeloproliferative neoplasms, polycythemia vera, primary myelofibrosis

## Abstract

Treatment with PEGylated interferon‐alpha2 (IFN) of patients with essential thrombocythemia and polycythemia vera induces major molecular remissions with a reduction in the *JAK2*V617F allele burden to undetectable levels in a subset of patients. A favorable response to IFN has been argued to depend upon the tumor burden, implying that institution of treatment with IFN should be as early as possible after the diagnosis. However, evidence for this statement is not available. We present a thorough analysis of unique serial *JAK2*V617F measurements in 66 IFN‐treated patients and in 6 untreated patients. Without IFN treatment, the *JAK2*V617F allele burden increased exponentially with a period of doubling of 1.4 year. During monotherapy with IFN, the *JAK2*V617F allele burden decreased mono‐ or bi‐exponentially for 33 responders of which 28 patients satisfied both descriptions. Bi‐exponential description improved the fits in 19 cases being associated with late *JAK2*V617F responses. The decay of the *JAK2*V617F allele burden during IFN treatment was estimated to have half‐lives of 1.6 year for the monoexponential response and 1.0 year in the long term for the bi‐exponential response. In conclusion, through data‐driven analysis of the *JAK2*V617F allele burden, we provide novel information regarding the *JAK2*V617F kinetics during IFN‐treatment, arguing for early intervention.

## Graphical abstract

Data‐driven mathematics of the kinetics of the JAK2V617F allele burden during treatment with IFN is presented. The half‐life of the JAK2V617F allele burden was predicted as 1.6 year, and 1.0 year in the long term, supporting early treatment with IFN.

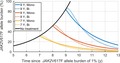

## 1 INTRODUCTION

The classic Philadelphia chromosome‐negative chronic myeloproliferative neoplasms (MPNs) encompass essential thrombocythemia (ET), polycythemia vera (PV), and primary myelofibrosis (PMF), including early prefibrotic myelofibrosis. These neoplasms arise due to an acquired stem cell insult with ensuing clonal myeloproliferation in the biological continuum from the early cancer stages (ET and PV) to the advanced myelofibrosis stage^1^ and ultimately leukemic transformation.^2^, ^3^

Molecular markers for MPNs include *JAK2*V617F, *CALR* ‐and *MPL*‐mutations. These mutations are the so‐called driver mutations whereas additional mutations (e.g., *ASXL1*, *TET2*) are frequently recorded in the more advanced disease stages with severe myelofibrosis.^4^, ^5^, ^6^, ^7^, ^8^, ^9^, ^10^, ^11^, ^12^ The *JAK2*V617F mutation associates with laboratory (hemoglobin level, leukocyte count, platelet count, CD34+ count, serum lactic dehydrogenase, and in vivo granulocyte and platelet activation) and clinical (pruritus, thrombosis, spleen size) outcomes^8^, ^13^, ^14^, ^15^, ^16^, ^17^, ^18^, ^19^, ^20^, ^21^, ^22^, ^23^, ^24^, ^25^ and prognosis.^13^, ^17^ Accordingly, a new concept of these diseases as a biological continuum from ET over PV to PMF has emerged, implying the *JAK2*V617F mutational load to reflect the tumor burden as assessed by a rising leukocyte count and increasing splenomegaly during disease progression toward myelofibrotic and leukemic transformation.^26^ However, this hypothesis on the biological continuum is still being debated.

Interferon‐alpha2 (IFN) has been used in the treatment of MPNs for about 30 years and several studies have convincingly demonstrated that this agent is safe and highly efficacious in normalizing elevated cell counts.^27^, ^28^, ^29^, ^30^, ^31^, ^32^, ^33^, ^34^, ^35^, ^36^, ^37^, ^38^, ^39^, ^40^, ^41^, ^42^, ^43^, ^44^, ^45^, ^46^, ^47^, ^48^, ^49^, ^50^, ^51^, ^52^ Indeed, prolonged treatment (about 5 years) may be followed by polyclonal hematopoiesis, normalization of the bone marrow and low‐burden *JAK2*V617F in a subset of patients, even being sustained for 2‐3 years after discontinuation of IFN.^36^, ^48^

These highly encouraging results have been the focus of increasing interest, since we may enter a new era with “Minimal Residual Disease” (MRD) as a novel treatment goal.^36^, ^44^, ^48^

Early treatment to reduce or eradicate the malignant clone is of paramount importance for achievement of MRD or cure in all cancers. However, in MPNs a “watch and wait” strategy is used in “low‐risk” patients allowing the malignant clone to expand with the inherent risk of increasing genomic instability, sub‐clone formation, resistance to treatment and disease progression from the early cancer stages (ET and PV) to the advanced metastatic cancer stage—myelofibrosis with bone marrow failure and ultimately leukemic transformation.

In recent years, the “watch and wait” strategy has been challenged by reports demonstrating the potential of IFN to induce MRD in an increasing number of patients.^36^, ^44^, ^48^ Furthermore, these studies also indicate that early treatment with IFN increases the chance of sustained hematological and molecular remissions. However, evidence for this statement is lacking.

As noted above, changes in the *JAK2*V617F allele burden before and during IFN‐treatment have been studied extensively, whereas the time‐scale kinetics of these changes still remain to be identified and described in detail.

In this study, we predict the *JAK2*V617F kinetics during IFN‐treatment through data‐driven analysis of serial *JAK2*V617F measurements in MPN patients receiving cytoreductive treatment with IFN only and patients being observed without cytoreduction. Evidence for tumor burden reduction through early intervention with IFN is provided, thereby challenging the “watch and wait” strategy commonly applied to low‐risk MPN patients.

## 2 METHODS

### 2.1 Study design

#### 2.1.1 Prospective study

Data were obtained from two different study populations. *JAK2*V617F observations during PEGylated r‐IFNα (IFN) monotherapy were obtained from 120 patients enrolled in the DALIAH trial (#EudraCT 2011‐001919‐31), which is an ongoing Danish multicenter prospective randomized open‐label phase III clinical trial comparing IFN with hydroxyurea in MPN patients. Enrollment began in February 2012 and was completed in July 2015. Patients are followed for five years. The DALIAH trial was approved by the Danish Regional Science Ethics Committee and by the Danish Medicines Agency.

The clinical characteristics of the patients used in later analysis is shown in Table 1.

**Table 1:** Prospective study. Baseline demographics and clinical characteristics of *JAK2*V617F positive patients from the DALIAH trial randomized to IFN. Only patients with four or more measurements of the *JAK2*V617F allele burden are included

Characteristics	ET	PV	Pre‐MF	PMF	Total
	(n = 15)	(n = 39)	(n = 5)	(n = 7)	(n = 66)
IFN type (IFNα‐2a/IFNα‐2b)	10/5	21/18	3/2	2/5	36/30
Age (y)	53 (43‐64)	64 (52‐69)	62 (59‐65)	64 (51‐65)	62 (51‐67)
Gender, male	6 (40)	20 (51)	3 (60)	6 (86)	35 (53)
History of major thrombotic event	0 (0)	12 (31)	1 (20)	2 (29)	15 (23)
*JAK2*V617F allele burden (%)	15 (10‐21)	44 (22‐62)	35 (19‐40)	51 (50‐88)	37 (19‐51)
Haematocrit (vol%)	44 (39‐47)	55 (48‐59)	45 (41‐45)	49 (37‐51)	49 (45‐55)
Haemoglobin (mmol/L)	9.0 (8.3‐9.7)	11.4 (10.0‐12.3)	9.2 (8.6‐9.5)	9.6 (7.2‐10.2)	9.9 (9.1‐11.8)
Platelets (×10^9^/L)	730 (626‐887)	538 (343‐670)	681 (667‐776)	460 (351‐611)	611 (413‐739)
White blood cells (× 10^9^/L)	8.9 (7.6‐11.9)	9.9 (8.4‐13.2)	9.2 (8.6‐9.5)	10.8 (5.5‐17.4)	9.7 (8.2‐12.9)
Plasma lactate dehydrogenase (U/L)	193 (164‐210)	229 (199‐304)	281 (181‐342)	367 (284‐643)	222 (189‐308)
Splenomegaly (≥13 cm by US)	2/9 (22)	11/26 (42)	1/4 (25)	7/7 (100)	21/46 (46)
Disease‐related symptoms^a^	8 (53)	24 (62)	1 (20)	3 (43)	36 (55)
Phlebotomy before enrolment	2 (13)	35 (90)	1 (20)	4 (57)	42 (64)
Low‐risk disease^b^	10 (67)	13 (33)	1 (20)	3 (43)	27 (41)

Note

Data are median (IQR) and n (%)

a Constitutional symptoms, microvascular disturbances and pruritus

b Age ≤ 60 y of age, platelets ≤ 1500 (×10^9^/L) and no prior major thrombosis

#### 2.1.2 Retrospective study

We retrospectively obtained information on *JAK2*V617F kinetics in six untreated (i.e., no cytoreductive therapy) MPN patients followed at the outpatient clinic at the Department of Haematology, Zealand University Hospital, Denmark. Four patients had previously received cytoreductive therapy with either r‐IFNα‐2a (Pegasys^®^) or r‐IFNα‐2b (PegIntron^®^) (n = 2) or monotherapy with both HU and r‐IFNα‐2a (n = 2) according to standard care, but had discontinued therapy due to intolerability (r‐IFNα‐2a: n = 2, HU: n = 2) and/or hematologic response in concert with a low JAK2V617F allele burden (r‐IFNα‐2a: n = 1, r‐IFNα‐2b: n = 1). Patient (A) discontinued treatment due to *JAK2*V617F < 1% for more than 1 year after r‐IFNα‐2b exposure and patient C discontinued r‐IFNα‐2a due to complete hematologic response in concert with a low JAK2V617F allele burden (6%). Two patients had not received any prior cytoreductive treatment. At the time of inclusion in the study all untreated patients had been off cytoreductive treatment for at least 0.5 months (median: 1.2 month; range 0.5‐5.3 months).

Patients were evaluated for enrollment when attending regular appointments between 1st of May 2018 and 15th of December 2018.

Written informed consent was provided from all patients according to the Declaration of Helsinki.

The clinical characteristics of the patients in the retrospective study are shown in Table 2.

**Table 2:** Retrospective study. Baseline demographics and clinical characteristics of *JAK2*V617F patients from the outpatient clinic at the time of first *JAK2*V617F measurement

Characteristics	ET	PV	Post‐PV MF	Total
	(n = 1)	(n = 4)	(n = 1)	(n = 6)
Age (y)	68	66 (59‐70)	75	68 (59‐75)
Gender, male	0 (0)	4 (80)	1 (100)	5 (83)
History of major thrombohemorrhagic event	0 (0)	3 (75)	0 (0)	3 (50)
Prior cytoreductive therapy	1 (100)	2 (50)	1 (100)	4 (67)
Hydroxyurea	1 (100)	0 (0)	1 (100)	2 (33)
r‐IFNα‐2a	1 (100)	1 (100)	1 (100)	3 (50)
r‐IFNα‐2b	0 (0)	1 (100)	0 (0)	1 (17)
Time off cytoreductive therapy before first *JAK2V617F* measurement (months)	0.5	1.2 (0.6‐1.8)	5.3	1.2 (0.5‐5.3)
*JAK2*V617F allele (%)	6	6 (0.7‐27)	93	8.5 (0.7‐96)

### 2.2 MPN diagnosis and eligibility

Eligibility criteria were age ≥ 18 years and a diagnosis of *JAK2*V617F positive Philadelphia chromosome negative MPN according the World Health Organization criteria.^53^ Patients from the DALIAH trial were all newly diagnosed or previously phlebotomized only, and all had evidence of active disease at enrolment. Active disease was defined by a requirement for phlebotomy, WBC > 10 × 10^9^/L or platelets > 400 × 10^9^/L in the absence of infection or inflammation, hypermetabolic symptoms ie weight loss > 10% within 6 months, night sweats, low‐grade fever for more than 2 weeks without signs of infection, pruritus, splenomegaly with symptoms, or previous thrombosis.

Eligible patients from the outpatient clinic studied retrospectively off cytoreductive treatment have been described above.

### 2.3 Intervention

DALIAH patients received monotherapy with either IFNα‐2a or r‐IFNα‐2b subcutaneously once weekly at a starting dose of 45 and 35 µg, respectively. Dose escalation was performed in a stepwise manner at pre‐defined time points in the absence of a complete hematological response (i.e., WBC > 10 × 10^9^/L or platelets > 400 × 10^9^/L) after 4 and 12 months and in the absence of a partial or complete molecular response according to the 2009 European LeukaemiaNet (ELN) criteria^54^ after 8 and 18 months. However, the IFN dose was de‐escalated by the treating physician to the highest tolerable dose in the event of drug‐related toxicity. Toxicity was graded according to the Common Terminology Criteria for Adverse Events (CTCAE) version 4.0 and IFN was discontinued in the event of grade 4 events or recurrent grade 3 events (See Supplementary Material E).

### 2.4 Molecular diagnosis and *JAK2*V617F

The *JAK2*V617F allele burden was accessed by a highly sensitive quantitative real‐time polymerase chain reaction (qPCR) on DNA from peripheral blood,^55^ which has been assessed as the European Reference Assay.^56^ In the DALIAH‐trial, *JAK2*V617F measurements were performed every three months the first year, once every six months the second year, and yearly thereafter until the end of the study. For patients followed in the outpatient clinic, the *JAK2*V617F allele burden was assessed according to the physician’s decision. All patients with four or more *JAK2*V617F samples were eligible for data‐driven analysis.

### 2.5 *JAK2*V617F allele burden development in untreated patients

Due to the expanding nature of malignant cells, the *JAK2*V617F allele burden in untreated patients is expected to increase exponentially. Hence, it is reasonable to assume that an exponentially increasing function can be fitted to data and an exponential growth‐rate for specific patients can be found.

To generalize from multiple patient‐specific growth‐rates, we calculated the mean of the growth‐rates, resulting in an expression of the growth on a population level. The expression describes the expected growth of the *JAK2*V617F allele burden in a larger population across multiple orders of magnitude, even if the estimate is made on a small sample of the population. Since the *JAK2*V617F allele burden varies at diagnosis, we shifted data in time such that the individual patient‐specific fits coalesced with the population‐level growth‐curve at the mean time of these observations. Data were then pooled into a single data‐set, and the exponential growth was estimated, see Table S5. All growth rates were found with MATLAB R2018a, using the least square fitting method *fit* included in the *curve fit* toolbox.

Of the six patients available for this analysis, one was excluded since all measurements were ≥90%, and thus above the point at which the growth is expected to be exponential. Note that this patient is the single post‐PV MF patient shown in Table 2. For two of the remaining patients, a low number of *JAK2*V617F measurements were available, due to which these were excluded in the initial part of the analysis. This leaves three remaining patients, referred to as patient A, B, and C.

### 2.6 *JAK2*V617F allele burden development during IFN monotherapy

To describe the *JAK2*V617F development in IFN treated patients, two simple descriptive models were fitted to the time series measurements. The first model is that of a monoexponential decay, i.e., of the form *Ae^−αt^* (with *A* and α being positive constants and *t* the time from treatment onset), while the second model features a bi‐exponential decay *Be^−βt^ − Ce^−γt^* (With *B*, *C*, *β* and *γ* being positive constants and *t* the time from treatment onset) with the requirement that the slope at treatment onset is equal to the slope found for the monoexponential growth. This reduces the number of independent parameters from four to three. The bi‐exponential model can be considered an extension of the mono‐exponential model with the bi‐exponential model describing a similar response but allowing for an initial *JAK2*V617F increase before the decay starts.

Since the bi‐exponential model has three parameters, patients with three or less *JAK2*V617F measurements were excluded. This reduced the number of datasets available from 120 to 66. We identified how well the models fit data by the adjusted *R* ^2^‐value, as described in supplementary material A. Thus, fits with an adjusted *R* ^2^‐value below a threshold of 0.6 were excluded from further analysis. The analysis for each of the two models was done independently.

## 3 RESULTS

### 3.1 *JAK2*V617F allele burden development in untreated patients

Raw data used to estimate the *JAK2*V617F allele burden development in untreated patients are depicted in Figures S72‐S76.

Three patients (A, B and C) received IFN treatment up to the date of the initial measurement.

**Figure 1:**
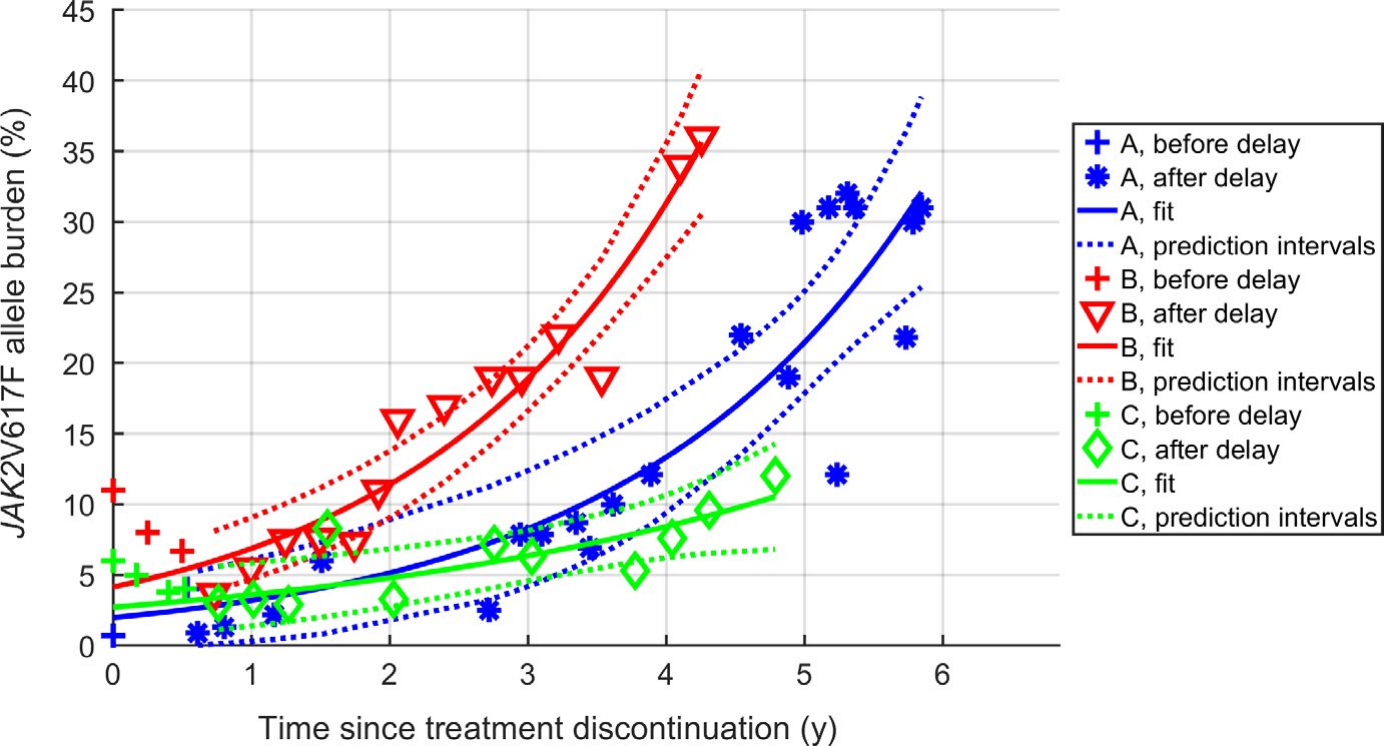
*JAK2*V617F allele burden development in untreated patients. Serial measurements for patients A, B and C with least‐square fits of exponential growth with 95% prediction intervals. Fits were based only on data after the initial delayed response

After IFN discontinuation, there was an initial delayed response in the *JAK2*V617F allele burden of approximately 200 days. Only data after the initial delayed response were used to determine the growth‐rates. Data are illustrated in Figure 1 as well as the least‐square fits of mono‐exponential growth to the data, with 95% prediction intervals.

An exponential growth‐rate implies a constant period of doubling. The periods of doubling found for patients A, B, and C are shown in Table S5, along with confidence intervals.

**Figure 2:**
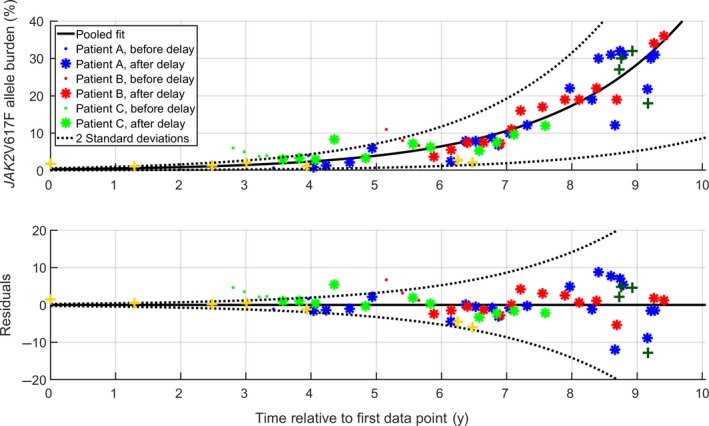
Exponential growth of the *JAK2*V617F allele burden. The top displays the exponential growth fit of the pooled data, while the bottom displays the relative residuals scaled with the size of allele burden. +‐marks indicate the two patients with very few *JAK2*V617F measurements. The initial delayed response is included as dots. Each subject is depicted with a specific color. Two standard deviations around the mean of the scaled residuals are shown

By pooling the data together, a new fit was made, yielding a general expression for the growth at population level. The resulting population level expression is shown in Figure 2 along with the pooled data and data for the two additional datasets which had only few *JAK2*V617F measurements. The additional datasets were timeshifted such that the mean of the *JAK2*V617F allele burden coalesced with the growth curve at the mean day of the dataset. While the two additional patients were not included in the fitting procedure, the data did not falsify the population level growth of the pooled data.

The period of doubling of the pooled data was found as 1.4 years (CI: 1.2 to 1.7 years). This implies that the allele burden grows from 0.01% to 1% in 9.3 years, while the growth from 1% to 33% takes 7.1 years. Therefore, detecting the *JAK2*V617F allele burden ≤ 1% allows for a much longer time‐window for detection and early therapeutic intervention before symptoms arise.

### 3.2 *JAK2*V617F allele burden development during IFN monotherapy

All 66 eligible data‐sets are depicted in the Figure S6 through S71.

Patient‐specific parameters for the fits, the goodness of the fits, and which model was the better descriptor are shown in Supplementary Material A.

To generalize the *JAK2*V617F kinetics, a threshold for the goodness‐of‐fit was chosen and estimates for the population‐level parameters were found, see Supplementary Material B.

Fits of 28 patients were deemed satisfactory for both response types (i.e., both mono‐ and bi‐exponential). For 14 of these patients, the bi‐exponential response was found to be the better fit for the patient data. Note that the goodness‐of‐fit measure chosen takes the complexity of the models into account, and as such the simpler monoexponential response was preferred when the models yielded almost identical results. Five additional patients had satisfactory fits for the bi‐exponential response type but not for the monoexponential. Thus, the bi‐exponential model was the best fit for 19 patients.

The datasets for the remaining 33 patients either featured responses not following any of the models or had no decay in the *JAK2*V617F allele burden and are hence considered nonresponders in the context of molecular response.

**Figure 3:**
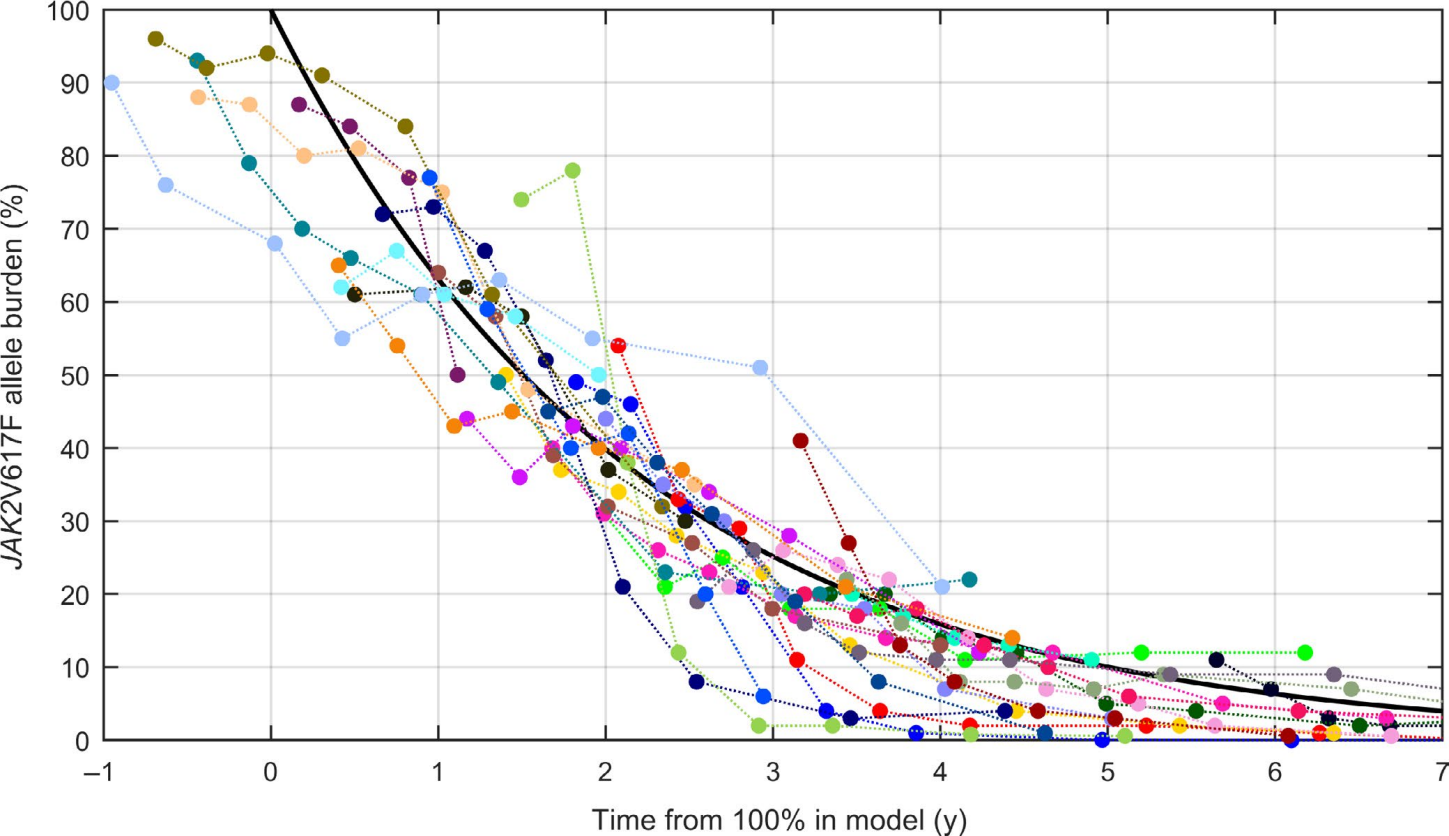
Population‐level decay rate. The population‐level decay rate of the mono‐exponential model is shown in black, with data for the 29 patients with the best goodness‐of‐fits. Note that data were shifted in time such that the mean of the *JAK2*V617F allele burden coincides with the main curve at the mean day of measurement

The monoexponential model using the population decay‐rate is shown in Figure 3, along with patient‐data for the patients for whom the goodness‐of‐fits were above the threshold.

The population decay rates found for the monoexponential model corresponded to a half‐life of 575 days, or 1.6 years. (95% CI of the decay rates yields half‐lives between 0.8 and 11.6 years).

For the monoexponential decay, the development was the same across all orders of magnitude, allowing for a single representative figure, Figure 3. Although the bi‐exponential model features an initial growth depending on the starting level, it has a long‐term behavior which is approximately monoexponential. This long‐term behavior corresponds to a half‐life of 1 year (CI: 0.2‐4.3 years).

### 3.3 Comparison of *JAK2*V617F allele burden development during early and late IFN monotherapy

**Figure 4:**
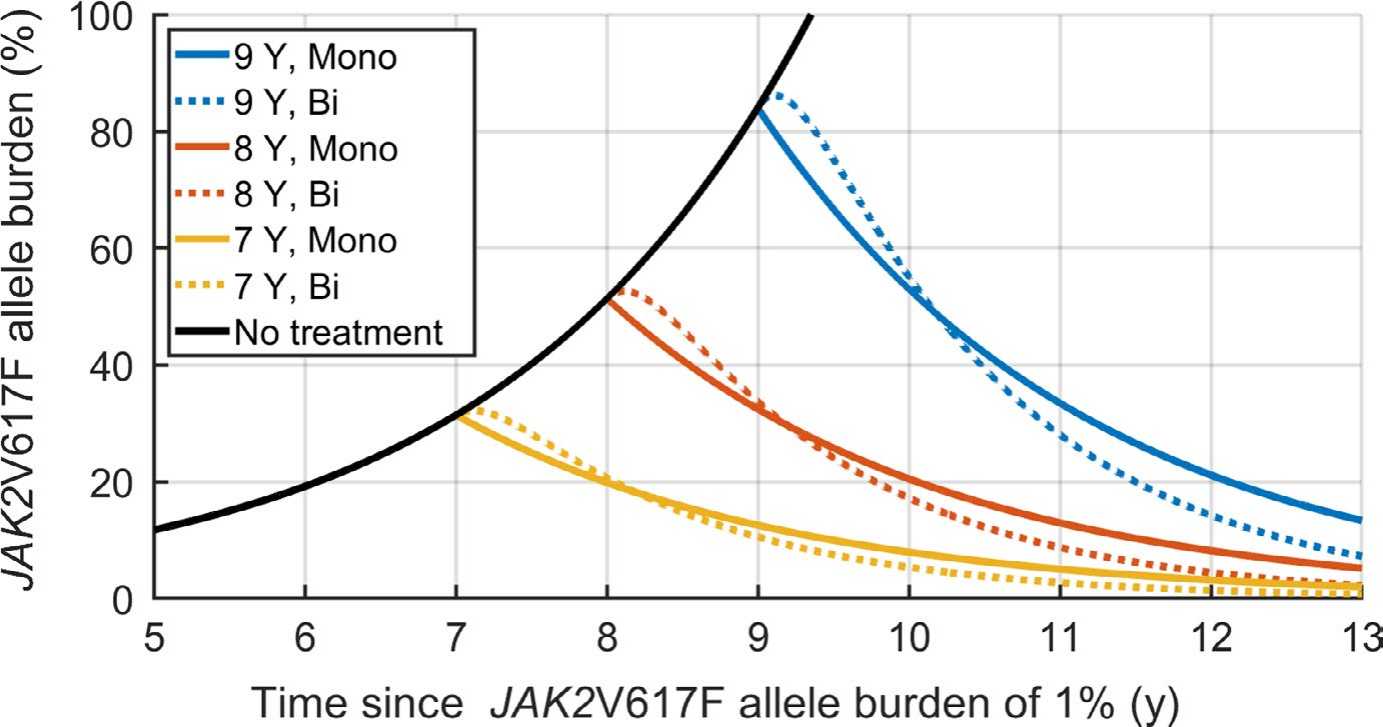
Prediction of *JAK2*V617F development during IFN treatment. *JAK2*V617F development over time estimated by either the mono‐exponential or bi‐exponential response (full lines: Mono‐exponential, dotted lines: bi‐exponential), with simulated treatment starting at 7, 8 and 9 y after 1% was reached

In silico treatment schemes can be considered using the population level models and the growth rates found. Figure 4 displays the increase and decrease in *JAK2*V617F allele burden, with initiation of IFN treatment 7, 8 and 9 years after the allele burden reached 1%. The figure shows both the monoexponential response as well as the bi‐exponential response at population level.

The figure suggests that the initial 6 months of IFN treatment are associated with minor or no decrease in the *JAK2*V617F allele burden for the bi‐exponential response. However, after a year, the *JAK2*V617F allele burden decreases faster than for the monoexponential response. As such, the efficacy of the treatment in decreasing the *JAK2*V617FV allele burden may be difficult to determine within the first year.

**Figure 5:**
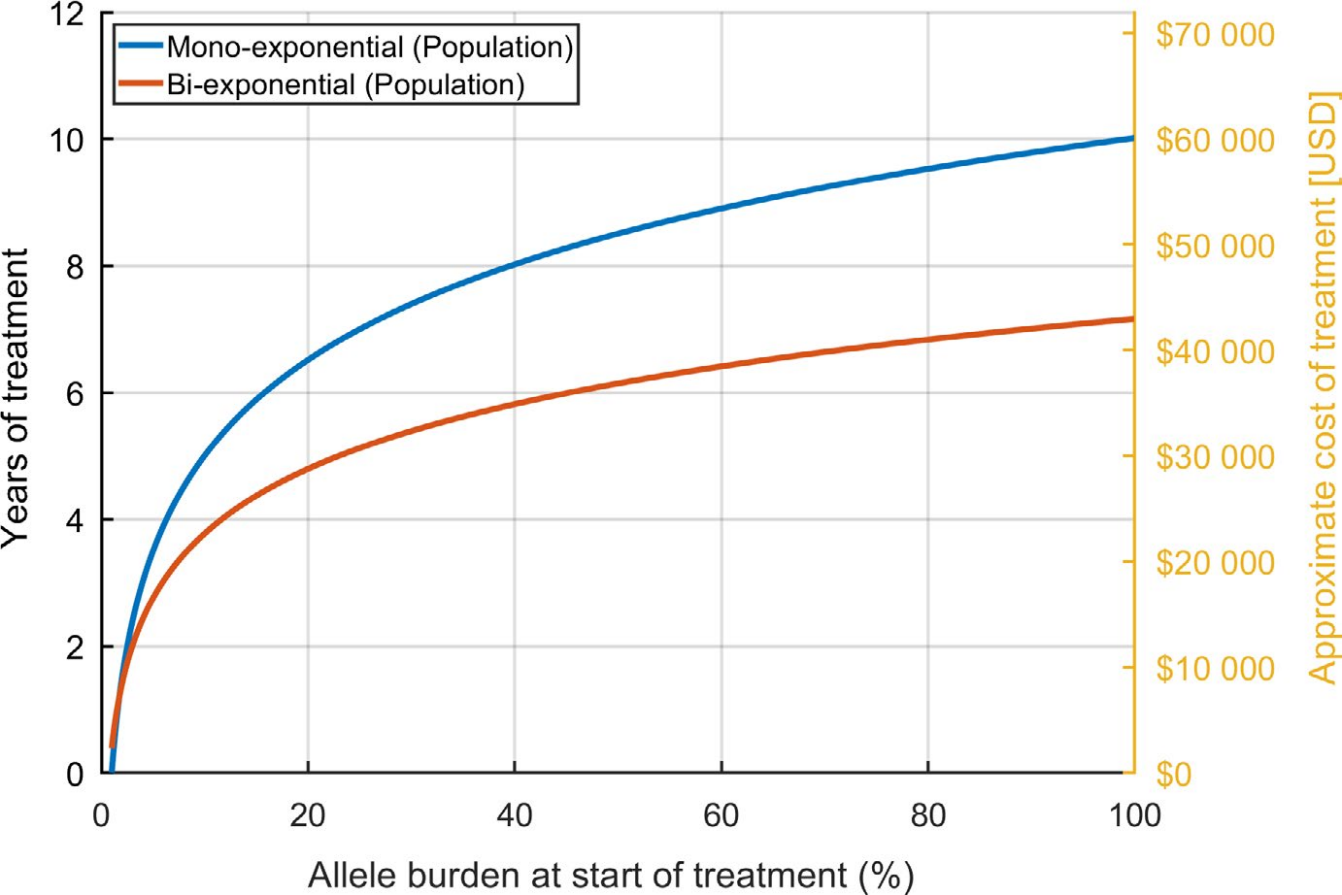
Estimated cost of IFN treatment. Years of treatment necessary to reach a *JAK2*V617F allele burden of 1%, if treatment is initiated at the allele burden shown on the first axis. The approximate monetary cost of IFN treatment (estimated price: $500 USD/month) is also included

From a specific baseline *JAK2*V617F allele burden, the population‐level responses can be used to estimate the duration of treatment necessary to achieve a *JAK2*V617F allele burden of 1%. In Figure 5 the duration of treatment necessary is illustrated, when treatment is initiated at a given *JAK2*V617F allele burden.

The monoexponential response features a longer treatment period necessary than the bi‐exponential response, when the initial *JAK2*V617F allele burden is greater than a few percent. As such, even if a patient has an initial increase in *JAK2*V617F allele burden as is the case for the bi‐exponential response, 1% will be reached faster than if the response was monoexponential.

Figure 5 also includes a rough estimate of the total medical expenses to IFN treatment (approximately $500 USD/month) based on the *JAK2*V617F allele burden at treatment onset. While relapse after treatment is still possible, the figure illustrates that the total cost of IFN treatment increases with the *JAK2*V617F allele burden at treatment onset due to the need for longer treatment duration to reach a specific *JAK2*V617F target value.

### 3.4 Time span of the *JAK2*V617F allele burden development

The growth of the *JAK2*V617F allele burden from 0.01% to 1% was found to span almost a decade, while further growth to 33% required approximately 7 years. If patients are screened for the *JAK2*V617F mutation on a regular basis, eg once every 10 years, a detection limit of 1% might miss the disease onset, since the *JAK2*V617F allele burden would exceed 33% before the next screening. Conversely, a detection limit of 0.01% could be expected to identify an allele burden below 1% during the 9.3‐year period, or it may grow to 1.4% 10 years after an allele burden of 0.01%. This also emphasizes the importance of methods to quantify the *JAK2*V617F allele burden down to low levels, in particular below 1%^.^

Although exponential growth may not be expected for low numbers of cells, extrapolating back to the time at which the initial mutation appeared (i.e., an allele burden corresponding to a single cell) yields a conservative estimate of the time span of the development before symptoms arise and MPN is diagnosed. The *JAK2*V617F allele burden for patients diagnosed with PV has previously been estimated as 33% (CI: 20‐40).^57^ We assume that the *JAK2*V617F allele burden in the peripheral blood and of the stem cells are similar. The total number of stem cells has been estimated to be between 11 200 and 22 400 cells,^58^ which is of order 10^4^. As such, an estimate of the allele burden at initial mutation is 10^−4^, ie one out of 10^4^. This implies a total time from initial mutation to allele burden of 33% of around 16.5 years (CI: 14.2‐19.5 years). This estimate of the timespan of MPN development is in agreement with the literature.^59^

**Figure 6:**
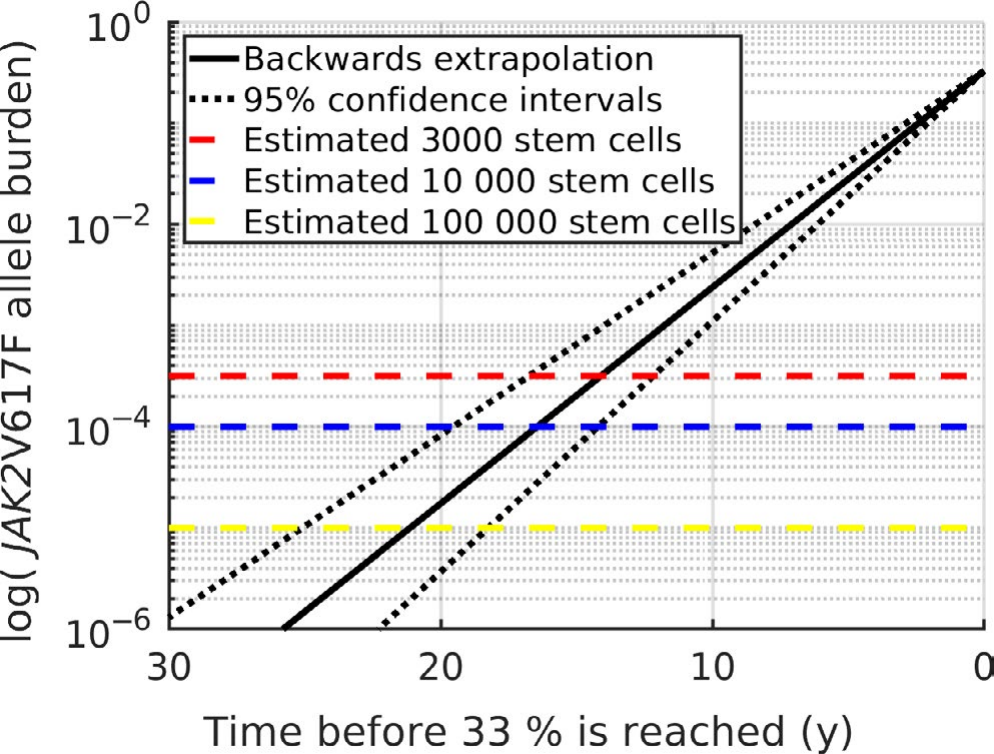
Backwards extrapolation to onset of initial mutation. The growth rate (and confidence intervals in dotted black) extrapolated back in time from 33%. The dashed lines mark the limits of 10^−3.5^, 10^−4^ and 10^−5^ as discussed in the text, in red, blue and yellow, respectively. Note the logarithmic *y*‐axis

Figure 6 shows the extrapolation from an allele burden of 33% backwards in time, with the growth‐rate found. The estimate is highly dependent on the total number of stem cells. If another estimate of the number of stem cells is used, the period before 33% is reached can be found from the graph, as well as the confidence intervals. Estimating a lower stem cell count of 3000^60^ leads to an allele burden of 10^−3.5^ at initial mutation, and the time of development would be approximately 14 years. Similarly, estimating a stem cell count between 50 000 and 200 000 as in a most recent paper^61^ leads to an initial allele burden around 10^−5^, and consequently a timespan of 21 years for the development of MPNs.

## 4 DISCUSSION

In recent years, the interest of using IFN in the treatment of MPNs has increased due to studies reporting long‐term treatment with IFN to be associated with MRD in a subset of patients as defined by sustained complete hematological remissions in concert with induction of low‐burden *JAK2*V617F and normalization of the bone marrow.^36^, ^44^, ^48^ Even in the IFN era of 2019 where several safety and efficacy studies have enrolled > 1000 patients during the last 30 years, the MPN scientific community still recommends patients with low risk disease to be observed without any cytoreductive treatment. Using this “watch and wait” strategy translation of common knowledge on cancer biology to MPNs is neglected, implying progression of any cancer without treatment. Early treatment with IFN has been claimed to be a prerequisite for obtaining remarkable results to prohibit clonal evolution before subclones and additive mutations evolve.^38^, ^40^, ^41^, ^62^, ^63^, ^64^, ^65^, ^66^, ^67^

The present study delivers novel information regarding the *JAK2*V617F kinetics during IFN‐treatment based upon unique serial *JAK2*V617F measurements from the DALIAH trial. Through this description of the *JAK2*V617F kinetics, predictions about the development of disease for specific patients can be made. In untreated patients (i.e., without cytoreductive treatment), the *JAK2*V617F allele burden was demonstrated to grow exponentially with doubling time of 1.4 years (CI: 1.2 to 1.7 years). During IFN treatment, the *JAK2*V617F development followed either a monoexponential or a bi‐exponential decay for a significant proportion of patients, with both models describing the development to a satisfactory extent for 28 patients. In a total of 33 patients, the bi‐exponential response was found to be the better descriptor of the development when adjusting for model complexity.

The individual *JAK2*V617F developments were combined into two models of the development on a population level. The population‐level model of the monoexponential decay showed a half‐life of the *JAK2*V617F allele burden of 1.6 years (CI: 0.8 to 11.6 years), while the long‐term behavior of the bi‐exponential decay displayed a half‐life of 1 year (CI: 0.2‐4.3 years).

We emphasize that since these population‐level models are based on a subset of patients who respond well to treatment, the models do not necessarily generalize to all patients. Determining the development for the poor responders remains an open problem as well as determining why there is a difference in the treatment response.

Analysis of the population‐level responses suggests that treatment schemes should extend longer than one year, as the efficacy of IFN treatment on the *JAK2*V617F allele burden cannot be determined after just one year. By comparing early and late treatment modalities, our results suggest that treatment outcome will improve if IFN therapy is initiated early, or will in any case lead to early identification of patient response type. Additionally, some patients had a significantly slower response to treatment compared to other patients. For these slow‐responders, our results show, that treatment should be initiated as early as possible, since a “watch and wait” strategy increases the time needed to obtain responses drastically for each day spent watching and waiting.

We and others have argued against the “watch and wait” strategy in low‐risk patients.^38^, ^40^, ^41^, ^62^, ^63^, ^64^, ^65^, ^66^, ^67^ Our study emphasizes the urgent need to rethink this approach and set new standards for treatment of patients with MPNs, implying normalization of cell counts in all patients using IFN from the time of diagnosis. In addition to the rationales provided by the results in our present study, several others are supportive of the early‐IFN‐intervention concept. Thus, cancer biology in general dictates that any cancer steadily evolves over time with expansion of the malignant clone, increasing genomic instability, subclone formation and ultimately metastasis. Fortunately, MPNs are slowly growing neoplasms which have several transitional stages in the biological continuum from the earliest cancer stages (eg *JAK2*V617F and CALR mutations in the background population as clonal hematopoiesis of indeterminate potential (CHIP)) to ET, PV, and the advanced cancer stage with myelofibrosis, bone marrow failure, and huge splenomegaly before terminal leukemic transformation. During this MPN‐biological continuum, the *JAK2*V617F mutation will steadily increase in those individuals who develop overt MPN in concert with an increase in the chronic inflammatory load that drives the malignant clone and likely fuels the development of additional mutations as well.^64^, ^65^, ^66^, ^67^ Thus, in this context, it is tempting to suggest that the total number of mutated cells present at any given time may be the driver of additive mutations. Indeed, the sum of the *JAK2*V617F allele burden over a period of time may provide a measure for the risk of additive mutations as it correlates with the number of mutated cells in the given the period. As shown in Supplementary Material C, this measure—as assessed by the *JAK2*V617F allele burden—increases exponentially with the time spent without treatment. Although speculative, this suggests that, after enough time has passed above a certain threshold, the potential risk of additive mutations may have the same exponential growth‐rate (and thus the same period of doubling) as the growth of the *JAK2*V617F allele burden before treatment is initiated. As such, although the exact risk cannot be determined, postponing treatment by 1.4 years will double the risk of an additive mutation. Further data‐driven mathematical studies on this potential association are needed, including results from next generation sequencing in IFN‐treated *JAK2*V617F positive MPN‐cohorts.

Treatment with IFN is associated with drop‐out rates of 20%‐30% and in some studies even up to 40% due to toxic side effects.^37^, ^38^, ^39^, ^40^, ^41^, ^42^, ^43^ It has been speculated whether intolerance to IFN is also dependent upon disease stage, implying more toxic side effects in the advanced myelofibrosis stage and less so in the early disease stages. If so, evidence for exponential growth with doubling time of 1.4 years further undermines the “watch and wait" strategy and adds to the rationales of early treatment with IFN.

Our results deliver important information about treatment duration with IFN to obtain deep molecular remissions and long‐lasting sustained remissions after drug discontinuation. The mathematical models presented serve as a novel platform for predicting IFN response in individual patients. More advanced mechanism‐based mathematical models are foreseen to allow for improved prediction and insight into IFN response.

Our study dictates that institution of IFN at the earliest time point possible may have important socio‐economic implications as well. By minimizing the risk of complications (thrombosis, hemorrhages or cancer), a huge economic burden due to hospitalizations is likely markedly reduced. Importantly, costs concerning rehabilitation after these complications are reduced as well. Another important consequence of early treatment with IFN is the outlook to achieve MRD and at this time point the possibility of discontinuation of IFN for several years (up to 3‐5 years) when the patient is feeling healthy with normal cell counts. Of note, our study also suggests that early treatment with IFN is cost‐effective, implying a shorter treatment period with IFN if treatment is instituted at the earliest time point possible.

Although our data are supportive of early intervention with IFN it is important to underscore that our data do not deliver the clinical proof for this early intervention recommendation. This proof can only be delivered by the demonstration of reduction in clinically relevant end‐points such as thrombotic events, rate of transformation to myelofibrosis and acute leukemia. Indeed, the demonstration of these hard end‐points would require long‐term follow up of large cohorts of patients treated with IFN and a well‐designed control group. This study has never been reported and will likely never be reported in these orphan diseases, where randomized studies are so difficult to conduct ‐ in particular with follow‐up times of decades rather than for instance 5 years, when taking into account that leukemic transformation in general is a late event, developing in the advanced myelofibrosis stage of MPNs. Importantly, the randomized Proud/Continuation‐PV Phase III Trials showed RopegIFN (Besremi®) to be associated with a clear benefit over control (ie hydroxyurea) in achieving significant higher maintenance rates of complete hematological remission (CHR) over the course of treatment and in showing a significant lower risk of losing CHR. Since CHR can be considered as surrogate for risk of thrombosis, RopegIFN may be an optimal treatment modality for managing risk of thrombosis.^70^

Our study has some limitations. Firstly, our estimate of the development of the *JAK2*V617F allele burden was based specifically on the data from three patients only (A, B, and C) and longitudinal *JAK2*V617F allele burden measurements in more patients might have substantiated and strengthened our findings of an exponentially growing pattern, which has not previously been described mathematically. Secondly, we did not include serial measurements of the *JAK2*V617F allele burden after discontinuation of hydroxyurea. Unfortunately, we have not such data in our cohort of patients. However, based upon current knowledge on the kinetics of the leukocyte and platelet counts after a few days off hydroxyurea treatment, displaying rapid increases in the cell counts to pretreatment levels, it is reasonable to assume that the *JAK2*V617F allele burden might similarly increase after hydroxyurea discontinuation. An increase in the *JAK2*V617F allele burden when treatment with hydroxyurea is terminated has been previously demonstrated.^71^

Third, although the findings in our study are supportive of early treatment with IFN, it does not deliver the definite proof, which would require a study, showing that early IFN‐treatment from the time of diagnosis influences hard clinical end‐points, such as risk of thrombosis and major bleeding, transformation to myelofibrosis and acute myeloid leukemia and ultimately survival. Hopefully, our DALIAH trial, from which our data in the present study have been retrieved, may provide such data within the next 10‐20 years.

In conclusion, data‐driven analysis is a novel tool for providing further evidence for the concept of early intervention with IFN in MPNs. Our observations also emphasize that starting treatment early allows for identification of patient responses. Understanding the kinetics of the *JAK2*V617F allele burden is highly valuable in guiding future clinical decisions about IFN‐treatment of the individual patient. In this context, our findings substantiate and put in perspective the urgent need of personalized medicine with IFN in MPN‐patients.

## CONFLICT OF INTEREST

HH has received a research grant from Novartis Oncology.

## AUTHOR CONTRIBUTIONS

Rasmus K. Pedersen: Formal analysis (lead), project administration (supporting), methodology (supporting), software (lead), visualization, writing – original draft, and writing – review and editing. Morten Andersen: Formal analysis (supporting), methodology (supporting), software (supporting), supervision (supporting), and writing – review and editing. Trine A. Knudsen: Investigation (lead), and writing – review and editing. Zamra Sajid: Writing – review and editing. Johanne Gudmand‐Hoeyer: Writing – review and editing. Marc J. B. Dam: Formal analysis (supporting), software (supporting), writing – review and editing. Vibe Skov: Investigation (supporting), methodology (supporting), writing – review and editing. Lasse Kjær: Investigation (supporting), methodology (supporting), writing – review and editing. Christina Ellervik: Writing – review and editing. Thomas S. Larsen: Investigation (supporting). Dennis Hansen: Data curation (lead), investigation (supporting). Niels Pallisgaard: Methodology (supporting). Hans C. Hasselbalch: Conceptualization (lead), funding acquisition (equal), methodology (supporting), investigation (supporting), supervision (supporting), validation (equal), and writing – review and editing. Johnny T. Ottesen: Conceptualization, funding acquisition (equal), methodology (lead), software (supporting), supervision (lead), validation (equal), and writing – review and editing.

## Data Availability

The data that support the findings of this study are available on request from the corresponding author. The data are not publicly available due to privacy or ethical restrictions.
