# Supplementary material for: Data‐driven analysis of JAK2V617F kinetics during interferon‐alpha2 treatment of patients with polycythemia vera and related neoplasms
Source: Cancer Med. 2020 Jan 28;9(6):2039–51. doi: 10.1002/cam4.2741 (PMC7064092; doi:10.1002/cam4.2741)
Supplement: Supplementary file 1 [file CAM4-9-2039-s001.pdf]

Supplementary material

"Data-driven Analysis of *JAK2*V617F Kinetics During Interferon-Alpha2 Treatment of Patients with Polycythemia Vera and Related Neoplasms"

A Values of fit-parameters

In this section we present the specific values of the parameters for the 64 fits to the patient-responses. The fits are shown visually in the supplementary D.
For reference, the functional forms of the two models will be shown here.
The mono-exponential decay has form:

$$f_m(t) = A \cdot e^{-\alpha t} \tag{1}$$

The bi-exponential model is defined somewhat differently than described in the main paper. This is done in the fitting procedure to ensure that some parameters are positive and that the slope at $t = 0$ was continuous. As such the functional form is:

$$f_b(t) = B \cdot \left(\frac{\beta^2 + c^2 + \nu}{c^2} \cdot e^{-\beta^2 t} - \frac{\beta^2 + \nu}{c^2} \cdot e^{-\left(\beta^2 + c^2\right)t} \right) \tag{2}$$

Where ν is the growth-rate before treatment is initiated.
To determine how well a given model fits a dataset a multitude of goodness-of-fit (GoF) measures can be used. We use the adjusted R-square, $\bar{R}^2$ values as given by:

$$\bar{R}^2 = 1 - \frac{SSE(n-1)}{SST(n-m)} \tag{3}$$

Where SSE is the sum of squared errors, SST is the total sum of square, n is the number of data-points and m is the number of coefficients fitted.
This measure is automatically calculated as part of the MATLAB `fit` procedure.
In Supplemental table 1 the parameters found are shown, along with the corresponding adjusted R^2 goodness-of-fit values.

B Distribution of fit-parameters

In this section we will present the distributions of the fit-coefficients presented in supplementary A.
Firstly, this section serves to illustrate the distributions of patient-responses. Secondly, to describe how the parameters for a population-level mono-exponential response and for a population-level bi-exponential response were determined. Before presenting the arguments for the choices of parameter-values, the specific values found is shown in Supplemental table 2.
As such, the mono-exponential response from equation (1), can be expressed numerically as:

$$f_m(t) = A \cdot e^{-0.46t} \tag{4}$$

With the growth-rate before treatment estimated as 0.49 year^{-1} (yielding a period of doubling of 1.41 years), the bi-exponential response from equation (2) can be stated as a numerical expression:

$$f_b(t) = B \cdot \left(1.32e^{-0.74t} - 0.32e^{-4.61t}\right) \tag{5}$$

By visual inspection of the fits shown in the supplementary D, a conservative threshold for the minimal $\bar{R}^2$ accepted as a good fit is chosen as $\bar{R}^2 \geq 0.6$.

Mono-exponential

A total of 28 fits are above the threshold for the mono-exponential fit. In figure 1 a histogram of the α parameter-values of all fits of the mono-exponential model with $\bar{R}^2$ above the threshold is shown. Additionally, the Gaussian distribution best fitting these values is shown in black, as well as the result of a Gaussian mixture modelling of the data with two Gaussian distributions, shown in dashed blue. The Gaussian mixture modelling was fitted using the `fitgmdist` MATLAB function included in the *Statistics and Machine Learning Toolbox*. The function also evaluates the Akaike Information Criterion (AIC) which is used to determine whether one or two Gaussians was the best description of the distribution.
The single Gaussian has a mean of 0.62 and a standard deviation of 0.39, while the two Gaussians has means 0.46 and 1.35, standard deviations 0.19 and 0.23, with mixture coefficients of 0.82 and 0.18 respectively.

From the AIC the sum of the two Gaussian distributions is determined to be the better descriptor of the distribution of the values.
The two Gaussian distributions split the α-parameters in two groups. Since the Gaussian with the lower mean is representative for the majority of the α parameter-values, the population-level mono-exponential decay expression is determined to have an α of 0.46. With a standard deviation of 0.19, this yields 95 % confidence intervals between 0.08 and 0.84. Note that the group with the higher mean consists of patients that responded very well to treatment, and as such, picking the lower groups as the population response allows for a conservative estimate of the efficacy of treatment.
Since the A parameter of the mono-exponential form is simply the *JAK2*V617F allele burden at the initial time, determining a representative value for the parameter is unnecessary for present purposes.
As the threshold of $\bar{R}^2$ greater than 0.6 might be considered to strict, we also include the histogram and distribution given a threshold of 0.3. The less strict threshold leads to 38 acceptable fits. While these values are worse fits to the given patient-responses, the distribution is found to be similar, and thus the previously found values should also be a representative value for the additional patients. Figure 2 shows the distributions of α for the lower threshold.

Bi-exponential

A total of 32 fits are above the threshold for the mono-exponential fit. Figure 3 shows two histograms of the β^2 parameter values of all fits of the bi-exponential model with $\bar{R}^2$ above 0.6. One histogram (bottom) shows the β^2 distribution, while the other (top) displays the distribution of $\log(\beta^2)$. Additionally, the Gaussian distribution best fitting the $\log(\beta^2)$ distribution is shown in black, as well as the result of a Gaussian mixture modelling of the data with two Gaussian distributions, shown in dashed blue. This fitting of the Gaussian mixture model was done to the logarithmic values, as the distribution is assumed to be log-normally distributed.
From the distribution on a logarithmic scale, the single Gaussian has a mean of -0.39, with standard deviation of 0.74. The mixed Gaussians has means -0.63 and 0.54 with standard deviations 0.62 and 0.31 and mixture coefficients of 0.79 and 0.21 respectively.
In the case of the bi-exponential model, using the AIC, the single Gaussian distribution is determined to be the better descriptor. As such we have $\log(\beta^2) = -0.39$ or $\beta^2 = 0.68$ as our population-level value for β^2. Since the standard deviation is 0.74, the corresponding 95 % confidence intervals are 0.15 and 2.97.
As in the mono-exponential case, we also present a figure of the distributions found given a less strict threshold, namely a threshold of $\bar{R}^2 \geq 0.3$. The less strict threshold leads to 37 acceptable fits. This is found in figure 4.
The values for the c^2 parameters are found to vary across many orders of magnitude. This is to be expected as there is almost no difference between low values of c^2 once a certain low threshold has been reached, and similarly for high values of c^2. As such we decide to use the median value of fits with $\bar{R}^2 > 0.6$ for the population value of the c^2 parameter, yielding a value of 3.87.
As in the mono-exponential case, the parameter B is determined by the *JAK2*V617F allele burden at the initial time.

C Risk of secondary cancers

In this section we will derive a mathematical expression relating the time at which treatment is initiated with the mutational load, or the risk of secondary cancers.
For patients experiencing exponential growth of some disease indicator (such as *JAK2*V617F allele burden), and with treatment resulting in exponential decay of the disease indicator, the disease indicator can be described as:

$$f(t) = \begin{cases} Ae^{\alpha t} & \text{for } t \leq \tau \\ Ae^{\alpha\tau}e^{-\beta(t-\tau)} & \text{for } t > \tau \end{cases} \tag{6}$$

where A is some arbitrarily low detection limit, α is the exponential growth rate before treatment, β is the exponential decay rate during treatment and τ is the time at which treatment starts. We will use units of days, such that τ is in days, while α and β is in days^{-1}, but any matching units (e.g. years and years^{-1}) could also be used.
Considering some initial day, $t = 0$, where a lower limit A of the indicator can be measured, i.e. $f(0) = A$, and some final day $t_f > \tau$ at which the same detection limit is reached again, i.e. $f(t_f) = A$, the mathematical problem now has boundary conditions.
Note that $f(t_f) = Ae^{(\alpha+\beta)\tau}e^{-\beta t_f}$ since $t_f > \tau$. Since we have defined t_f such that $f(0) = f(t_f)$, this implies that $t_f = \frac{\alpha+\beta}{\beta}\tau$.

The number of days of treatment need to reach A is given as $t_f - \tau = \tau\left(\frac{\alpha+\beta}{\beta} - 1\right) = \frac{\alpha}{\beta}\tau$. Thus is it clear that for $\alpha > 0$ and $\beta > 0$, the days of treatment needed to reach A increases with τ.
Denoting the integral of $f(t)$ from $t = 0$ to $t = t_f$ as $F(t)$ we find:

$$F(t) = \int_0^{t_f} f(t)dt = A\int_0^{\tau} e^{\alpha t}dt + Ae^{(\alpha+\beta)\tau}\int_{\tau}^{t_f} e^{-\beta t}dt \tag{7}$$

$$= A\frac{e^{\alpha\tau}}{\alpha} - \frac{A}{\alpha} - \frac{A}{\beta} + A\frac{e^{\alpha\tau}}{\beta} \tag{8}$$

$$= A\left(e^{\alpha\tau} - 1\right)\left(\frac{1}{\alpha} + \frac{1}{\beta}\right). \tag{9}$$

For $\alpha\tau \ll 1$ we have $e^{\alpha\tau} - 1 \approx \alpha\tau$, and the expression is linear in τ. For $\alpha\tau \gg 1$, the expression is approximately $Ae^{\alpha\tau}\left(\frac{1}{\alpha} + \frac{1}{\beta}\right)$. As such the rate of doubling for large $\alpha\tau$ is $\frac{\log(2)}{\alpha}$.
If the disease indicator $f(t)$ is proportional to the number of mutated cells, there is a proportionality constant, M, relating $f(t)$ to the number of mutated cells at any given time. The integral of $Mf(t)$ for all time yields the accumulated number of mutated cells per day. Since $\int Mf(t)dt = MF(t)$, the average number of mutated cells per day is found to also double with rate $\frac{\log(2)}{\alpha}$. Assuming cells having a constant probability of dividing at any given time, the average number of division of mutated cells is given as $MrF(t)$ where r is the rate of division. Denoting this as

$$D(\tau) = MrA\left(e^{\alpha\tau} - 1\right)\left(\frac{1}{\alpha} + \frac{1}{\beta}\right). \tag{10}$$

If every division of mutated cells has a given probability of resulting in a second mutation, $D(\tau)$ is a measure of this probability. The doubling rate of $D(\tau)$ is also approximately $\frac{\log(2)}{\alpha}$ for $\alpha\tau \gg 1$ as was the case for $f(\tau)$. Given $\alpha \approx 0.0013$ and assuming $\tau \gg \frac{1}{\alpha} \approx 770$ days, we see that the risk of second mutations double approximately every 1.4 years treatment is postponed.

D Figures of fits

Data for all patients are shown in Supplemental figures 6 through 71 together with the two fit-types as well as an extrapolated growth following the exponential growth determined in the main paper. All figures are shown twice, with a linear y-axis of the left hand side and with a logarithmic y-axis on the right hand side.
Data is shown as black stars, the bi-exponential fit as a full red line, the mono-exponential fit as a dashed blue line and finally the extrapolated growth is shown in dotted black.

E Randomisation, criteria and IFN dosage

E.1 Randomisation, DALIAH

Patients $>$ 60 years of age:
I:I:I randomization to either Hydroxyurea, r-IFNα-2a, or r-IFNα-2b at starting dose according to dose-level 0.
Patients $\leq$ 60 years of age:
I:I randomization to either r-IFNα-2a, or r-IFNα-2b at starting dose according to dose-level 0.

E.2 Inclusion and exclusion criteria

Inclusion criteria

- Age $>$ 18 years of age at the time of signing the informed consent.
- ET, PV, Pre-MF or hyperproliferative PMF (WBC $> 10 \cdot 10^9$/l or platelets $> 400 \cdot 10^9$/l) according to the WHO criteria.
- No previous cytoreductive treatment with HU or IFN. However, HU was allowed from time of diagnosis to randomization. Previous phlebotomized PV patients were eligible.
- Active disease defined as WBC $> 10 \cdot 10^9$/l or platelets $> 400 \cdot 10^9$/l in the absence of infection or inflammation, need of phlebotomy, constitutional symptoms i.e. weight loss $>$ 10% within 6 months, night sweats, subfebrilia (temp. $>$ 38°C for more than 2 weeks without signs of infection), pruritus, symptomatic splenomegali, or previous thrombosis.

- Ability to comply to study visits and requirements.

Exclusion criteria

- Pregnant or lactating females
- Females of childbearing potential (FCBP) had to undergo pregnancy testing and the result had to be negative
- Inadequate or lack of acceptance to use adequate contraceptive method by FCBPs (oral contraceptives, intra uterine device, implant, transdermal patch, vaginal ring, depot injection). Infertile patients were exempted from using contraceptive methods. To be considered infertile the patients had to be surgically sterilized (vasectomy, bilateral tubectomy, hysterectomy, or ovariectomy) or post menopausal defined as amenorrhea < 12 months at time of inclusion)
- History of another malignancy within five years of enrolment (except basal cell carcinoma of the skin)
- Eastern Cooperative Group Oncology Status ≥ 3
- Serum creatinine $> 2 \times$ the upper limit of the normal range (ULN)
- Total bilirubin 1.5 $>$ ULN
- Plasma ALAT 3 $>$ ULN
- History of psychiatric disorder (depression diagnosed by psychiatrist)
- Autoimmune disorder
- Uncontrolled hyperthyroidism or hypothyroidism
- Severe cardiac disease (New York Heart Association Functional Class III or VI)
- Severe myelosuppression
- Chronic hepatitis infection with decompensated liver cirrhosis or recent (within 6 months) immunosupressive therapy except corticosteroids
- Epilepsy or other severe CNS disorders
- Hypersensitivity to recombinant interferon or hydroxyurea or excipients to these drugs

E.3 IFN dose-escalation

See Supplemental figure 5

E.4 IFN dose levels

See Supplemental table 3

E.5 IFN interruption or discontinuation due to adverse events

Adverse events were graded according to the Common Terminology Criteria for Adverse Events (CTCAE) version 4.0. Before IFN administration it was ensured that the platelet count $> 100 \cdot 10^9/\text{l}$, neutrophilic count $> 1 \cdot 10^9/\text{l}$ and that haemoglobin level > 6.2mmol/l. A maximum treatment interruption period of 6 months was allowed. Reasons for exclusion from the study included:

- Adverse events
- Other disease
- Non-compliance
- Exclusion criteria fulfilled
- Abnormal biochemistry
- Administrative problems
- Treatment interruption > 6 months

- Death
- Other reason

See Supplemental table 4 for details.

Supplemental tables and figures

	Best fit	Mono, α	Mono, A	Mono, $\bar{R}^2$	Bi, β	Bi, c	Bi, B	Bi, $\bar{R}^2$
Patient 1	Bi	1.058	54.784	0.935	1.520	-0.020	49.414	0.989
Patient 2	Mono	1.274	54.571	0.968	1.161	5.099	53.998	0.964
Patient 3	Mono	0.282	27.568	0.774	0.531	1603.103	27.569	0.729
Patient 4	Mono	0.743	10.257	0.453	0.864	1331.683	10.270	0.344
Patient 5	Mono	1.306	10.577	0.722	-1.143	1589.876	10.578	0.629
Patient 6	Mono	0.101	24.404	0.406	-0.318	2599.590	24.404	0.258
Patient 7	Mono	0.060	57.595	-0.302	0.244	1473.537	57.595	-1.604
Patient 8	Mono	0.497	37.614	0.977	0.705	3064.888	37.614	0.973
Patient 9	Mono	0.623	49.608	0.976	0.790	2929.200	49.608	0.972
Patient 10	Bi	0.670	21.859	0.937	0.923	2.004	20.338	0.958
Patient 11	Mono	0.036	31.287	-0.092	-0.191	1891.624	31.287	-0.310
Patient 12	Bi	0.109	45.810	0.359	0.725	0.001	38.369	0.685
Patient 13	Mono	0.724	45.122	0.966	0.881	3.816	43.946	0.962
Patient 14	Mono	0.619	65.174	0.967	0.808	4.524	64.024	0.962
Patient 15	Mono	0.203	0.492	-0.279	0.912	0.040	0.432	-0.901
Patient 16	Mono	0.135	24.569	0.346	-0.368	2652.808	24.569	0.237
Patient 17	Mono	0.439	19.544	0.962	-0.663	1356.620	19.544	0.943
Patient 18	Bi	0.026	2.561	-0.130	-0.611	0.012	2.145	0.057
Patient 19	Mono	0.379	91.802	0.965	0.616	2783.436	91.802	0.960
Patient 20	Mono	0.036	28.677	-0.241	0.189	2622.892	28.679	-0.655
Patient 21	Bi	0.754	84.352	0.870	1.296	-0.059	72.808	0.958
Patient 22	Bi	-0.192	6.911	0.479	0.393	0.001	6.236	0.528
Patient 23	Bi	1.402	85.293	0.869	1.728	-0.018	77.430	0.947
Patient 24	Bi	0.183	18.070	0.099	0.912	0.084	15.842	0.221
Patient 25	Mono	-0.001	56.522	-0.167	0.016	7.469	29.714	-3.670
Patient 26	Mono	-0.228	39.814	0.993	-0.000	1.150	38.668	0.740
Patient 27	Bi	-0.009	26.094	-0.122	-0.137	1.448	23.144	0.061
Patient 28	Mono	0.028	13.602	-0.073	0.167	1433.696	13.602	-0.252
Patient 29	Bi	-0.000	56.102	-0.167	-0.377	0.798	47.523	0.802
Patient 30	Bi	0.545	27.707	0.803	1.129	0.012	23.865	0.951
Patient 31	Bi	0.252	11.978	0.343	0.866	-0.062	9.971	0.544
Patient 32	Bi	-0.181	24.447	0.451	0.506	-0.027	22.628	0.640
Patient 33	Bi	0.288	46.089	0.757	0.925	0.001	38.567	0.846
Patient 34	Mono	0.485	6.502	0.270	1.324	-0.038	6.222	0.180
Patient 35	Bi	0.461	91.655	0.627	1.282	-0.000	85.871	0.736
Patient 36	Bi	0.509	21.030	0.940	0.812	1.720	19.018	0.949
Patient 37	Mono	0.305	13.391	-0.270	0.552	1300.860	13.391	-1.540
Patient 38	Bi	0.275	94.828	0.839	0.683	1.204	83.198	0.897
Patient 39	Mono	-0.347	21.328	0.233	-0.015	1.181	22.905	-1.290
Patient 40	Bi	0.320	67.526	0.655	1.037	-0.000	61.195	0.929
Patient 41	Bi	0.118	55.335	0.132	0.678	1.098	51.638	0.646
Patient 42	Mono	0.556	18.684	0.685	0.746	1330.142	18.687	0.622
Patient 43	Mono	0.330	62.178	0.939	0.574	2577.088	62.178	0.927
Patient 44	Mono	0.484	10.624	0.580	0.696	1209.771	10.625	0.495
Patient 45	Mono	-0.023	91.113	0.379	0.001	21.156	93.622	-0.507
Patient 46	Mono	-0.132	3.378	0.028	-0.001	4.843	3.767	-0.685
Patient 47	Mono	0.457	20.498	0.344	-0.677	1455.069	20.503	0.181
Patient 48	Mono	0.032	18.545	-0.169	0.180	2004.221	18.545	-0.462
Patient 49	Bi	0.262	105.049	0.769	0.908	-0.026	88.827	0.933
Patient 50	Bi	0.148	65.971	0.640	0.506	1.967	62.528	0.913
Patient 51	Mono	0.184	84.548	0.798	0.429	2490.317	84.548	0.764
Patient 52	Mono	0.262	36.321	0.302	-0.512	2619.741	36.325	0.163
Patient 53	Mono	0.301	20.813	0.688	0.570	3.125	20.007	0.653
Patient 54	Mono	0.125	36.188	0.380	-0.353	2653.729	36.189	0.255
Patient 55	Mono	0.445	43.563	0.577	-0.667	1564.166	43.567	0.436

Patient 56	Mono	0.219	27.316	-0.142	0.468	2595.645	27.316	-0.713
Patient 57	Bi	1.729	41.436	0.992	1.379	4.718	41.006	0.992
Patient 58	Mono	0.786	78.216	0.910	0.908	4.687	76.992	0.882
Patient 59	Mono	0.042	13.932	-0.236	-0.540	-0.883	12.792	-0.311
Patient 60	Mono	0.041	47.478	-0.194	0.202	1645.729	47.478	-0.791
Patient 61	Mono	0.125	50.756	-0.069	0.353	2649.928	50.756	-1.138
Patient 62	Mono	-0.268	12.669	0.470	-0.184	0.001	11.721	0.447
Patient 63	Bi	0.677	51.990	0.888	1.255	0.018	45.273	0.988
Patient 64	Mono	-0.105	54.436	0.670	0.014	1.185	51.497	0.394
Patient 65	Mono	-0.177	29.655	-0.214	0.000	1.623	29.320	-1.281
Patient 66	Mono	-0.019	0.110	-0.249	0.830	-0.014	0.111	-0.426

Supplemental table 1: All parameter-values as well as the goodness-of-fits for the 64 fits.

	α	A	
Mono-exponential	0.46 (CI: 0.08, 0.84)	N/A	
	β^2	c^2	B
Bi-exponential	0.74 (CI: 0.15, 2.97)	3.87	N/A

Supplemental table 2: Values found for the parameters of the two models.

Dose level	r-IFNα-2a	r-IFNα-2b
-3	35 μg/3 weeks	45 μg/3 weeks
-2	35 μg/2 weeks	45 μg/2 weeks
-1	35 μg/10 days	45 μg/10 days
0	35 μg/week	45 μg/week
+1	50 μg/week	90 μg/week
+2	96 μg/week	135 μg/week

Supplemental table 3: IFN Dose levels

**Category**	**Adverse event**	**IFN**
Non-haematological toxicity (CTCAE v. 4)	Grade 1 and 2	Unchanged dose if possible plus symptomatic treatment of side effects
	Grade 3	Interrupt. Restart at a dose level lower when decrease to grade 1 toxicity or lower
	Grade 4	Discontinue
Investigations (CTCAE v. 4)	ALAT $>$ 3.0 $\times$ ULN (grade 2)	Interrupt IFN. Restart once ALAT $\leq$ 3.0 $\times$ ULN (grade 1) at a dose level lower.
	Creatinine $>$ 1.5 $\times$ baseline ULN (grade 2)	
Endocrine disorders (CTCAE v. 4)	Hyperthyroidism during rIFNα treatment	Discontinue.
	Hypothyroidism during rIFNα treatment	Continue. Start thyroid hormone replacement
Depression (ICD-10 criteria)	Mild depression	First 4-8 weeks after onset of symptoms: Continue. Evaluation every 2 weeks.<br>- If recovery: Resume regular visit schedule<br>- If unchanged: Continue evaluation every 2 weeks<br>- If aggravation: See "moderate and severe depression"
	Moderate depression	First 4-8 weeks after onset of symptoms: Decrease rIFNα to a dose level -1 or -2. Evaluation once weekly;<br>- if recovery: Continue with dose level -1 or -2<br>- if unchanged: Decrease dose level and consider evaluation by psychiatrist<br>- if aggravation: See "severe depression"
	Severe depression	Discontinue. Evaluation by psychiatrist

Supplemental table 4: IFN interruption or discontinuation due to adverse events

Patient	Period of doubling (years)
A	1.5 (CI: 1.1, 2.2)
B	1.4 (CI: 1.2, 1.7)
C	2.5 (CI: 1.5, 6.0)
Pooled	1.4 (CI: 1.2, 1.7)

Supplemental table 5: Periods of *JAK2* V617F doubling for three patients (A, B and C) with confidence intervals (CI), as well as the period of doubling for the pooled data.

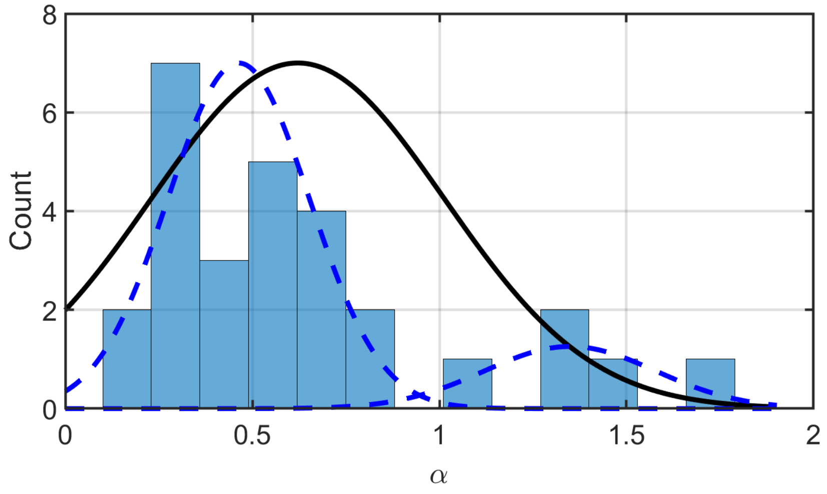

Supplemental figure 1: Distribution of α for the mono-exponential fits, for the 28 fits with an adjusted R^2 value greater than 0.6. The single Gaussian is shown in full black lines, while the dashed blue lines show the distribution following two Gaussians.

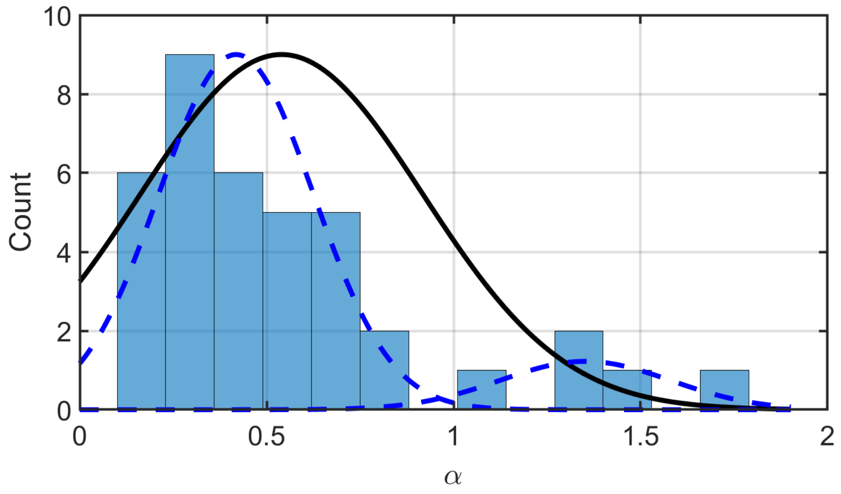

Supplemental figure 2: Distribution of α for the mono-exponential fits, for the 38 fits with an adjusted R^2 value greater than 0.3. The single Gaussian is shown in full black lines, while the dashed blue lines show the distribution following two Gaussians.

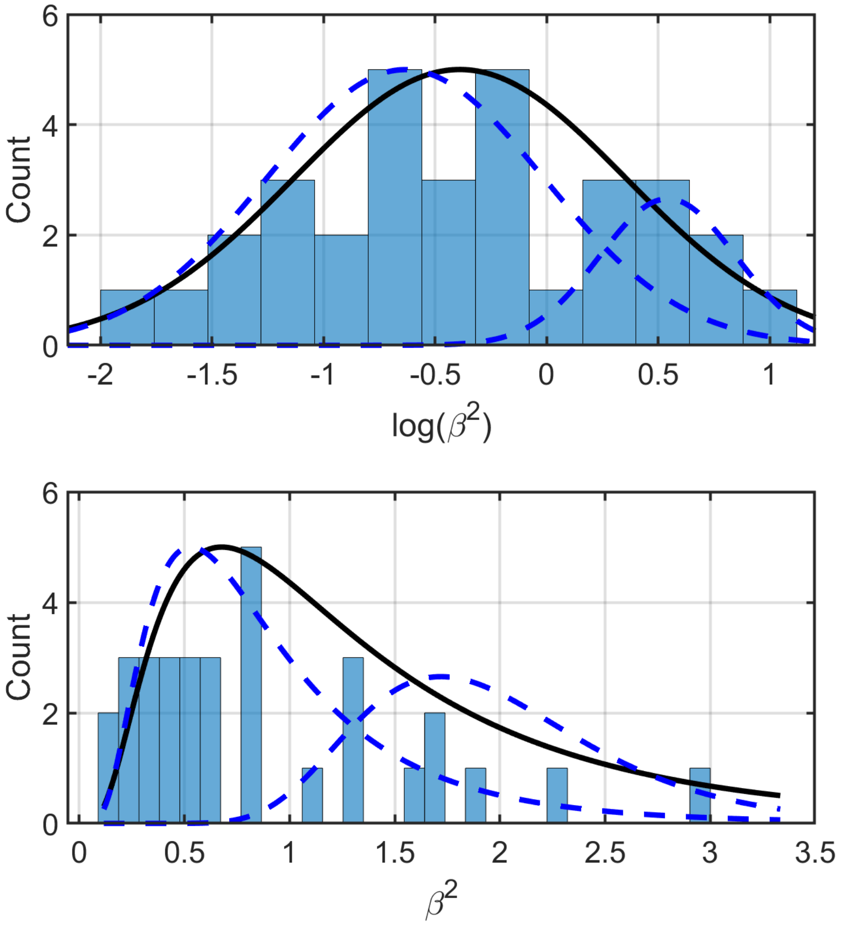

Supplemental figure 3: Distribution of β^2 for the bi-exponential fits, for the 32 fits with an adjusted R^2 value greater than 0.6. Top: Logarithmic first axis, bottom: Linear first axis. The single Gaussian is shown in full black lines, while the dashed blue lines show the distribution following two Gaussians.

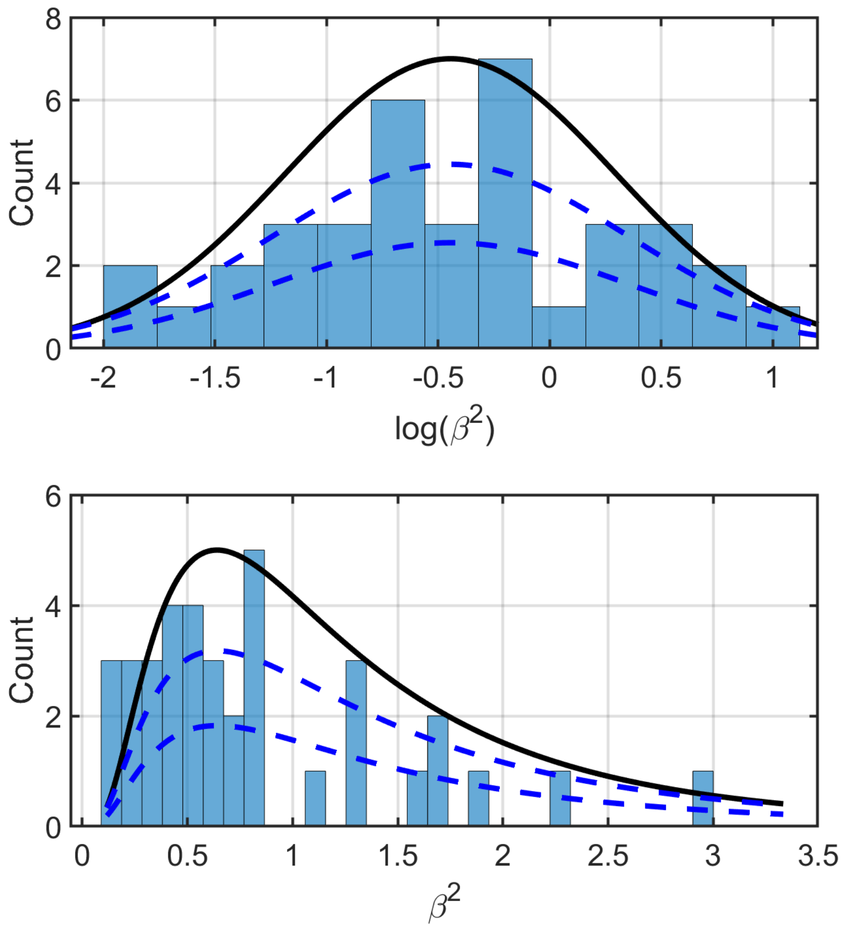

Supplemental figure 4: Distribution of β^2 for the bi-exponential fits, for the 37 fits with an adjusted R^2 value greater than 0.3. Top: Logarithmic first axis, bottom: Linear first axis. The single Gaussian is shown in full black lines, while the dashed blue lines show the distribution following two Gaussians.

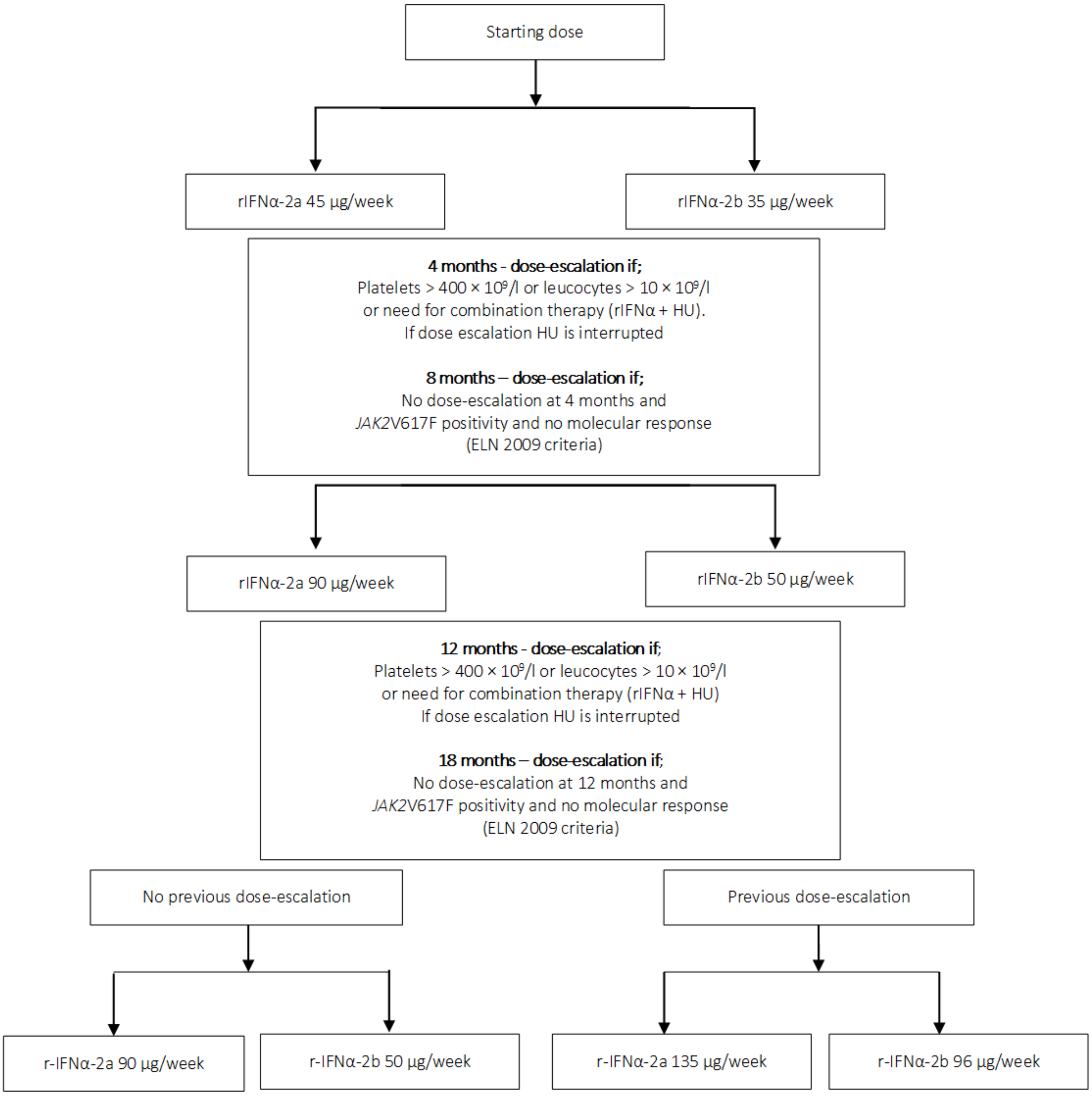

Supplemental figure 5: Chart for IFN dose-escalation

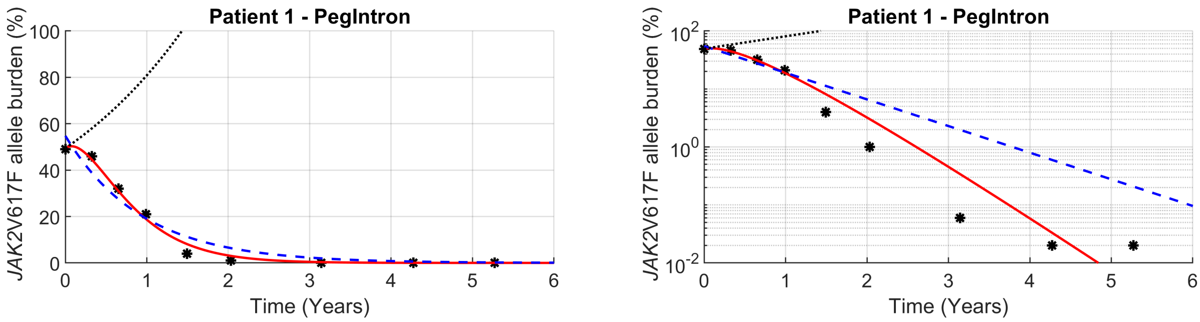

Supplemental figure 6: Data and model fits for patient 1. Data is shown as black stars, mono-exponential fit is shown as a dashed blue line and the bi-exponential fit is shown a full red line. An extrapolation of continued exponential growth is shown in a dotted black line. The right-hand figure displays the same as the left-hand figure, but on a logarithmic y-axis. On a logarithmic y-axis, the mono-exponential decay appears as a straight line, allowing for a simple visual check of the decay-rates.

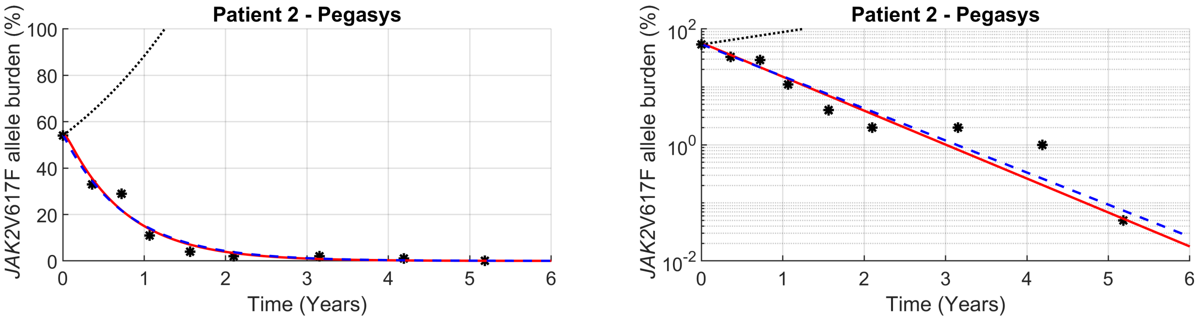

Supplemental figure 7: Data and model fits for patient 2, see legend of Supplemental figure 6

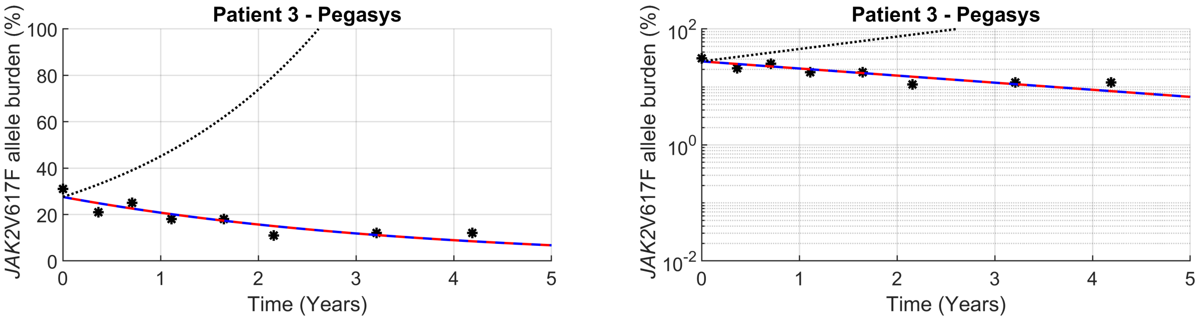

Supplemental figure 8: Data and model fits for patient 3, see legend of Supplemental figure 6

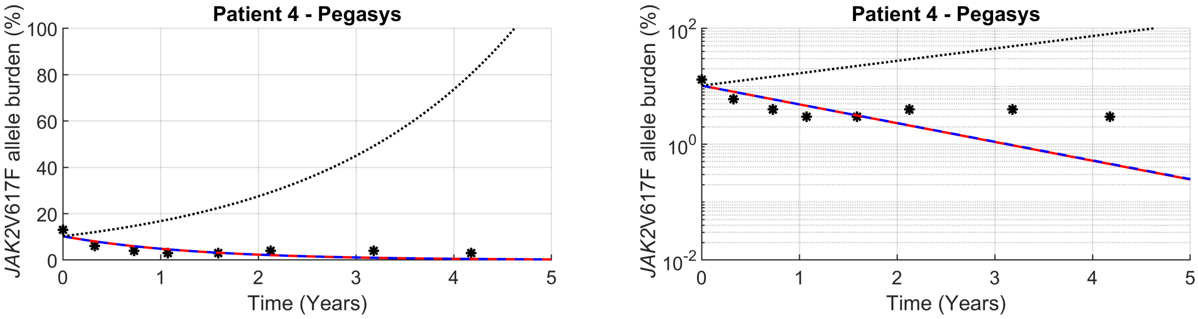

Supplemental figure 9: Data and model fits for patient 4, see legend of Supplemental figure 6

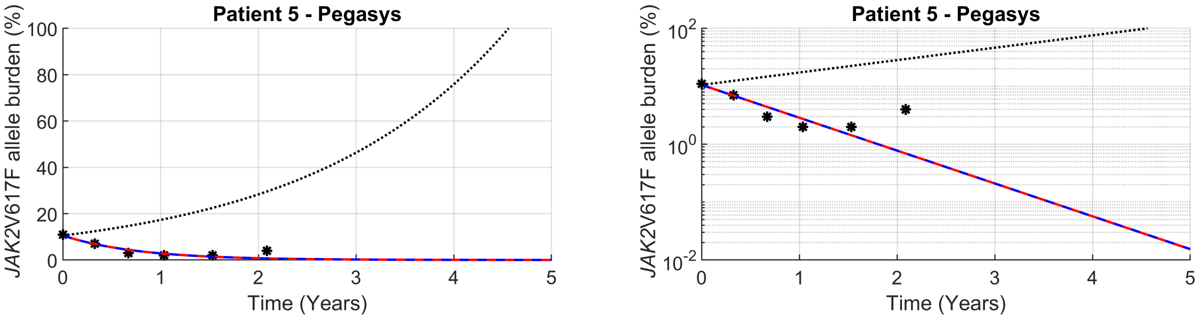

Supplemental figure 10: Data and model fits for patient 5, see legend of Supplemental figure 6

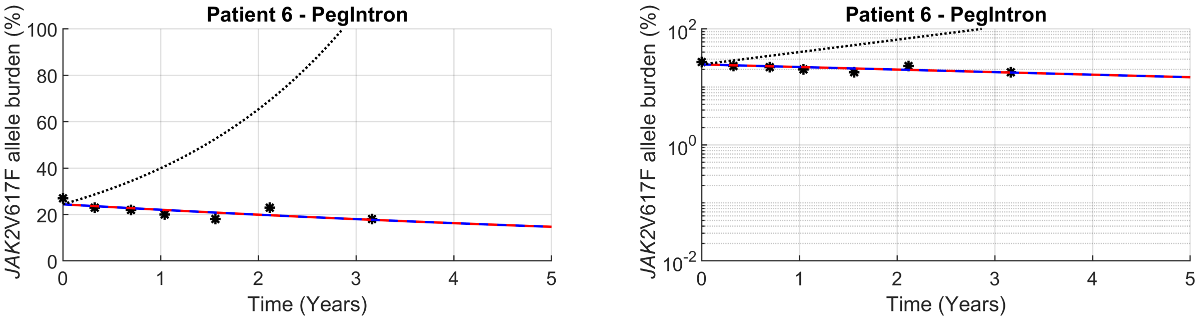

Supplemental figure 11: Data and model fits for patient 6, see legend of Supplemental figure 6

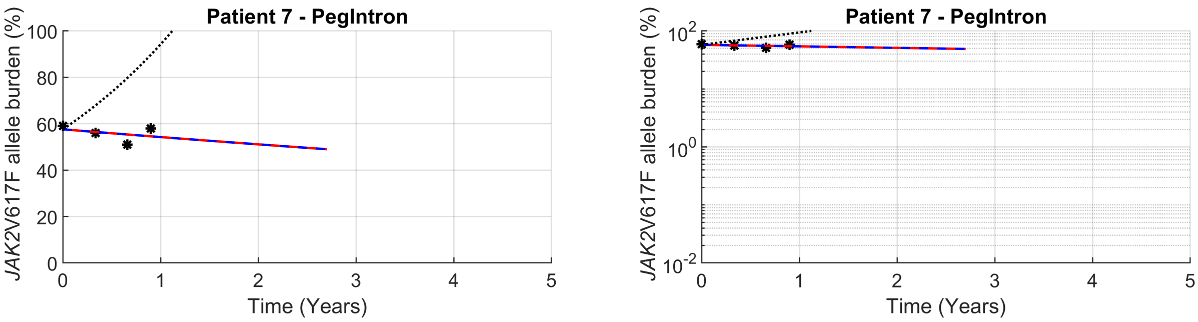

Supplemental figure 12: Data and model fits for patient 7, see legend of Supplemental figure 6

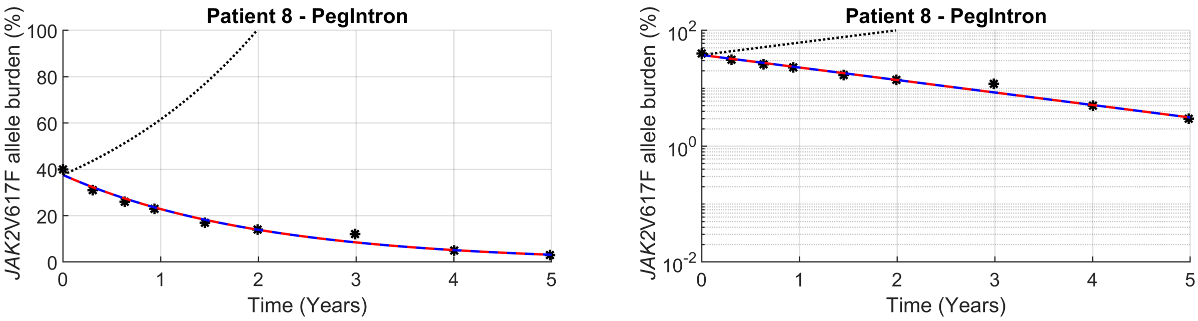

Supplemental figure 13: Data and model fits for patient 8, see legend of Supplemental figure 6

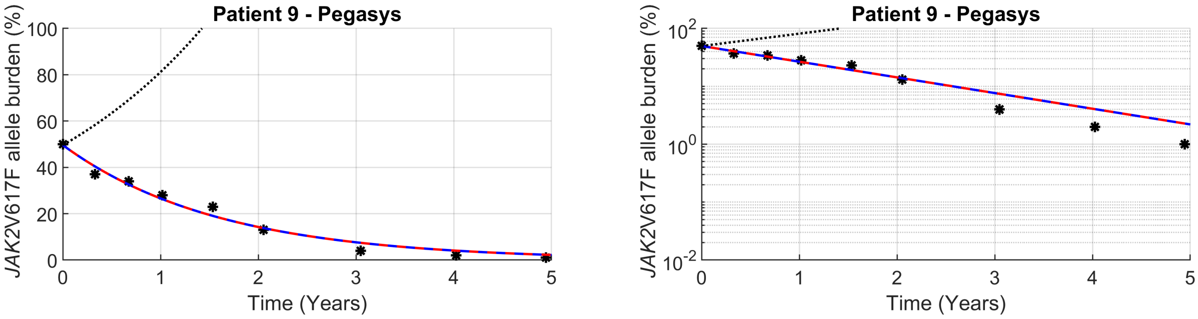

Supplemental figure 14: Data and model fits for patient 9, see legend of Supplemental figure 6

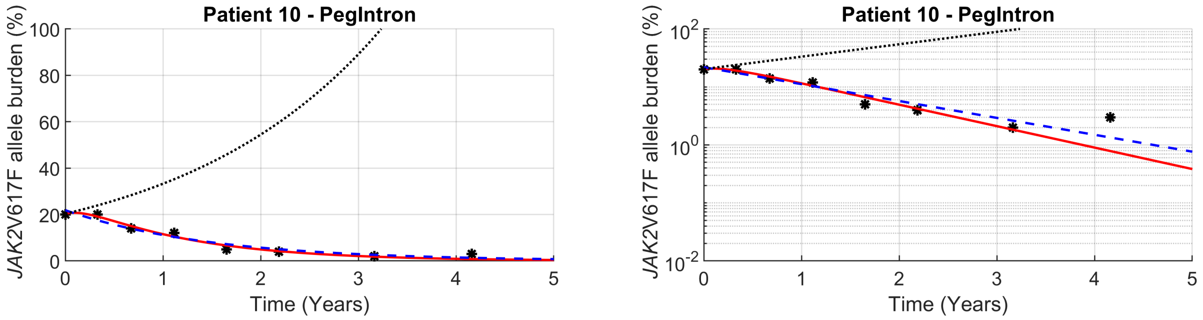

Supplemental figure 15: Data and model fits for patient 10, see legend of Supplemental figure 6

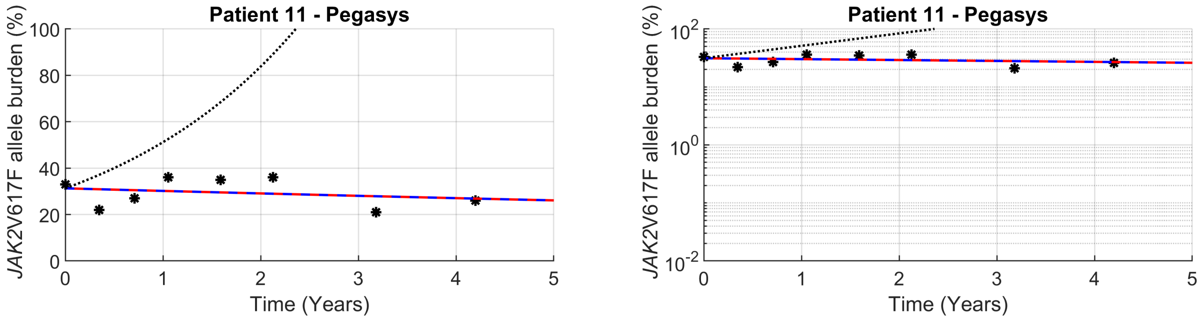

Supplemental figure 16: Data and model fits for patient 11, see legend of Supplemental figure 6

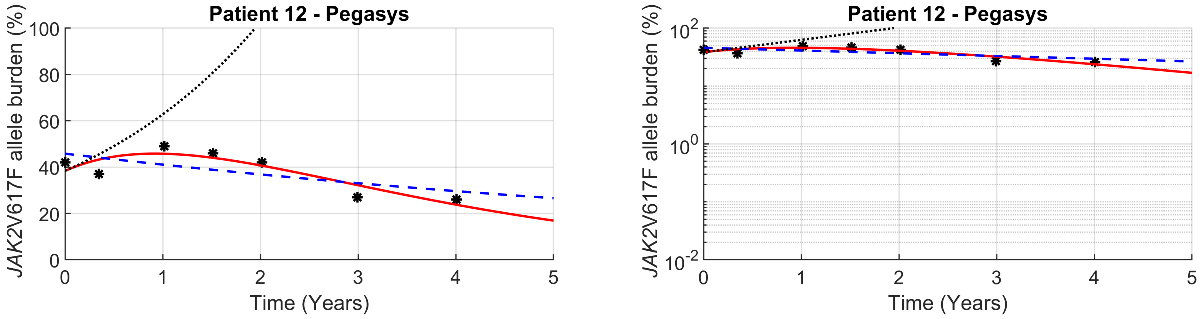

Supplemental figure 17: Data and model fits for patient 12, see legend of Supplemental figure 6

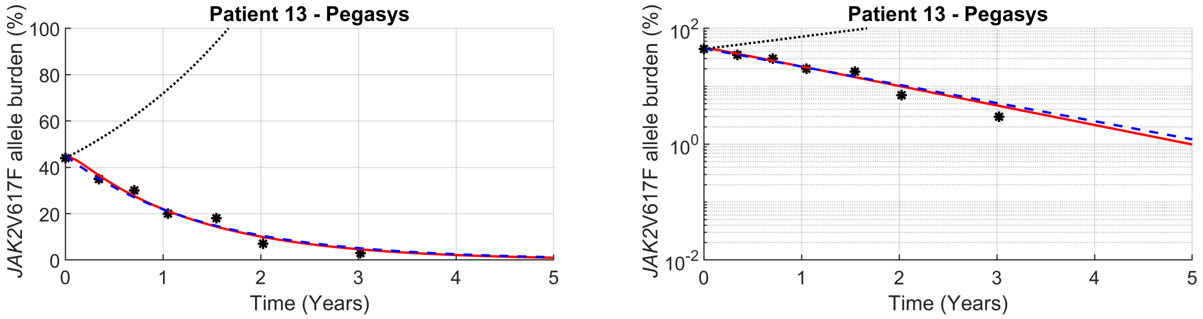

Supplemental figure 18: Data and model fits for patient 13, see legend of Supplemental figure 6

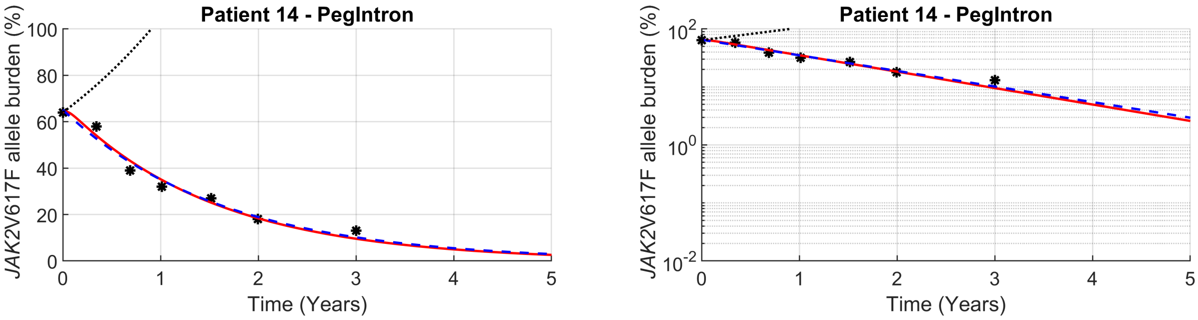

Supplemental figure 19: Data and model fits for patient 14, see legend of Supplemental figure 6

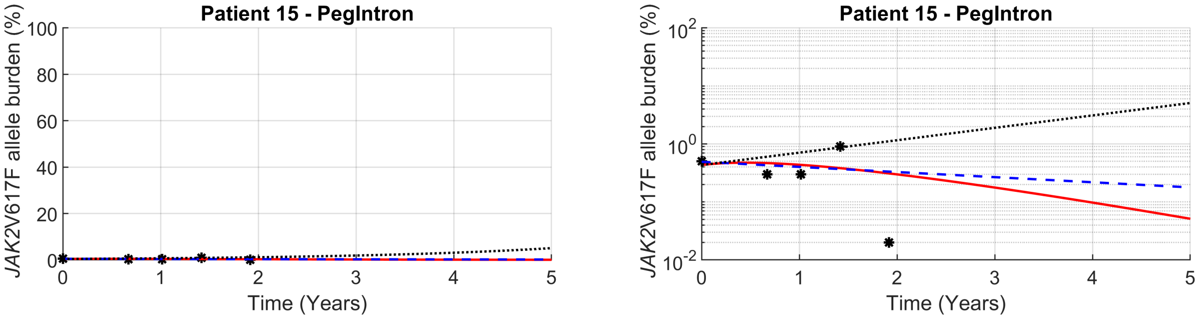

Supplemental figure 20: Data and model fits for patient 15, see legend of Supplemental figure 6

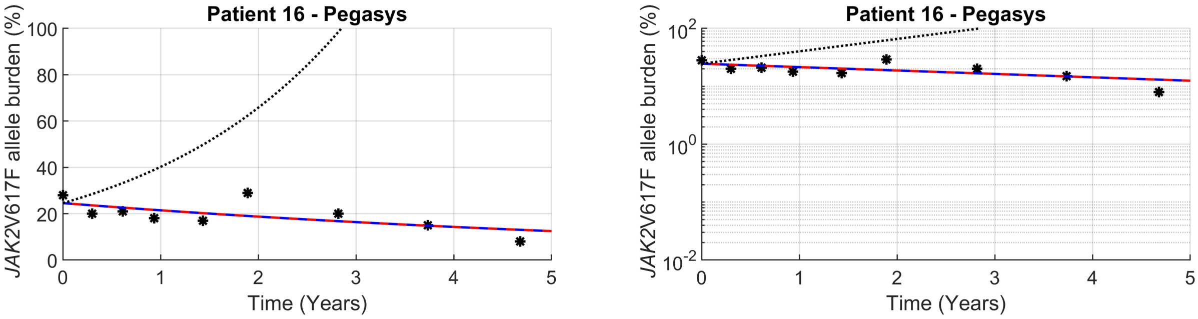

Supplemental figure 21: Data and model fits for patient 16, see legend of Supplemental figure 6

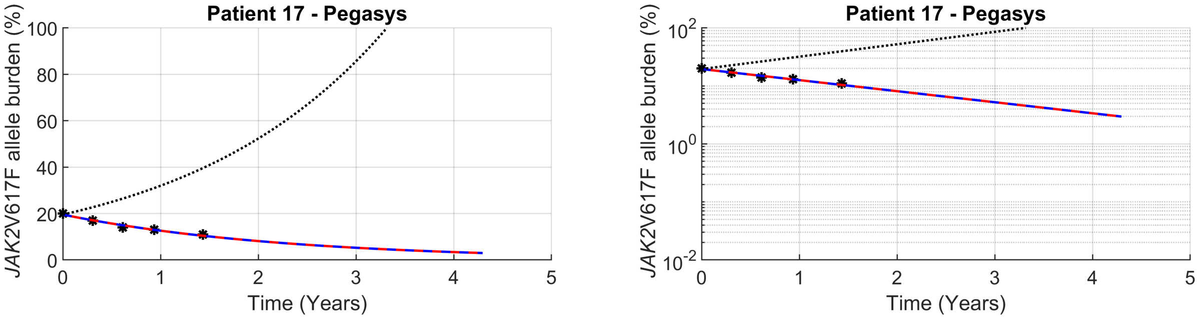

Supplemental figure 22: Data and model fits for patient 17, see legend of Supplemental figure 6

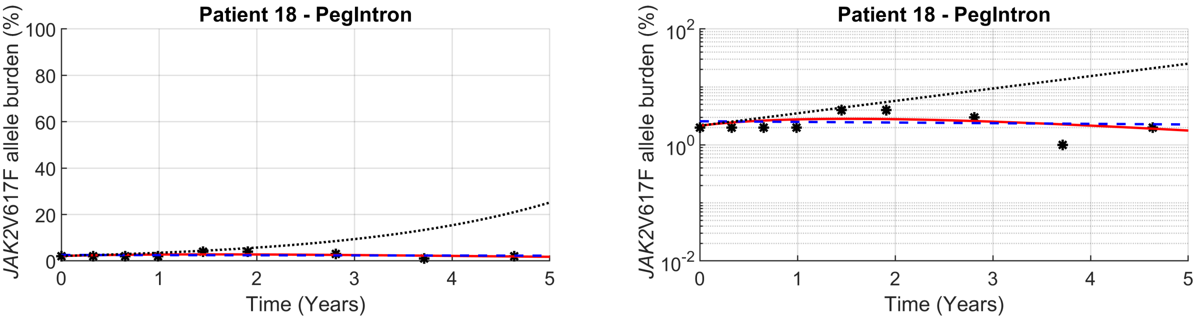

Supplemental figure 23: Data and model fits for patient 18, see legend of Supplemental figure 6

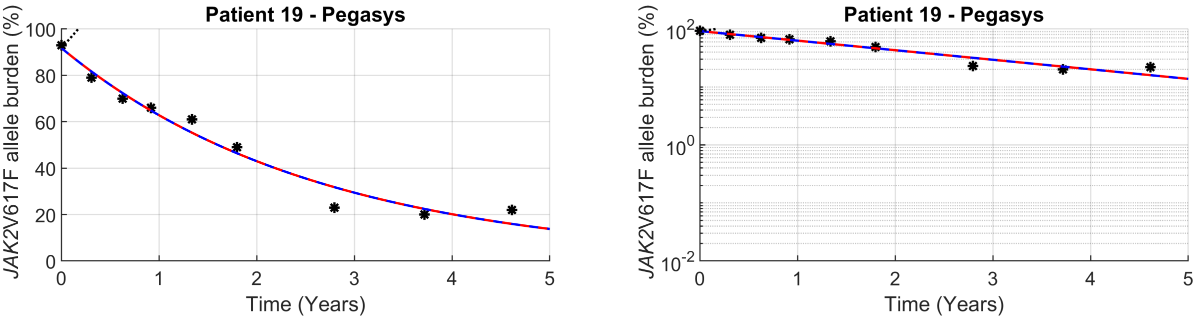

Supplemental figure 24: Data and model fits for patient 19, see legend of Supplemental figure 6

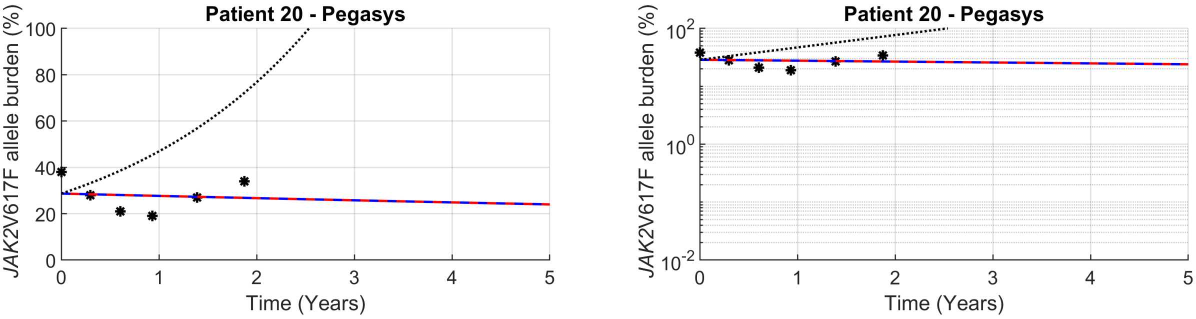

Supplemental figure 25: Data and model fits for patient 20, see legend of Supplemental figure 6

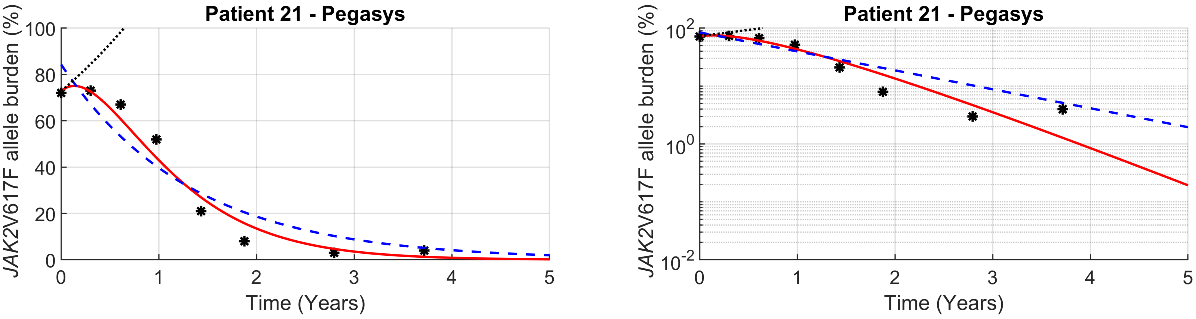

Supplemental figure 26: Data and model fits for patient 21, see legend of Supplemental figure 6

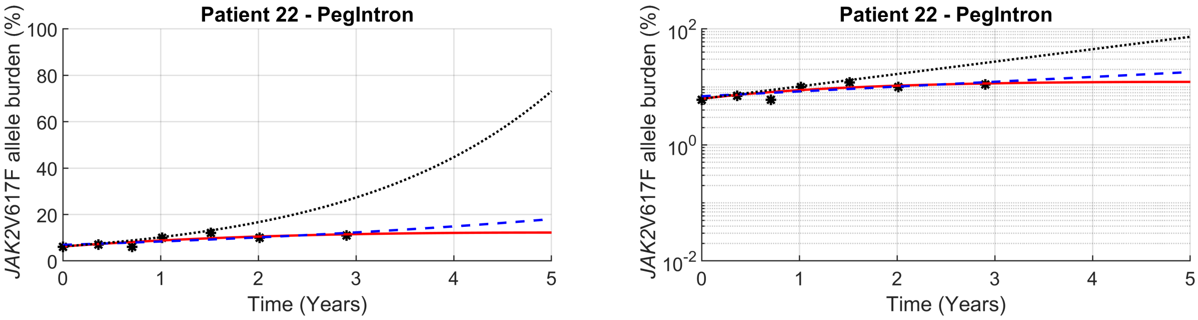

Supplemental figure 27: Data and model fits for patient 22, see legend of Supplemental figure 6

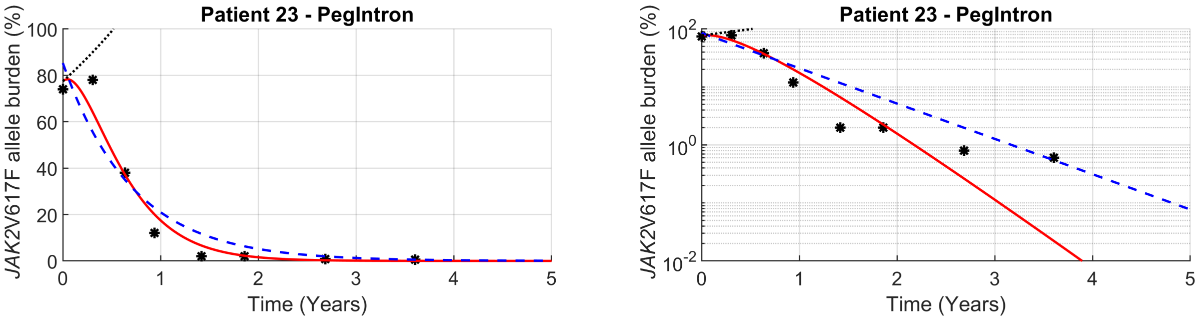

Supplemental figure 28: Data and model fits for patient 23, see legend of Supplemental figure 6

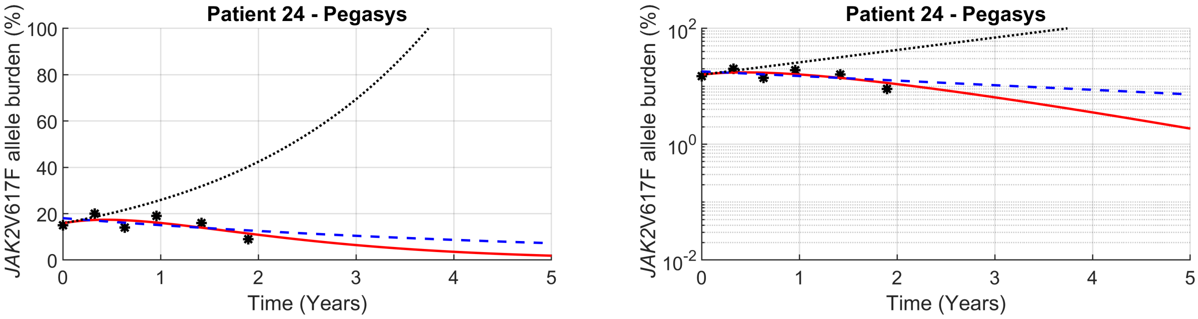

Supplemental figure 29: Data and model fits for patient 24, see legend of Supplemental figure 6

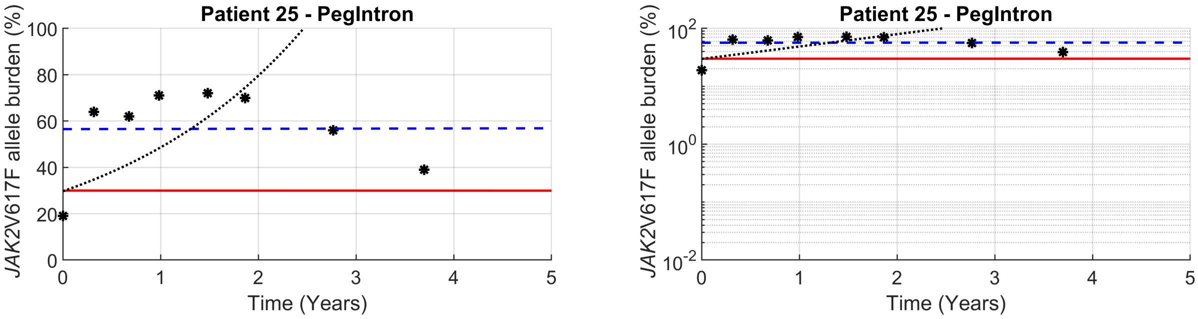

Supplemental figure 30: Data and model fits for patient 25, see legend of Supplemental figure 6

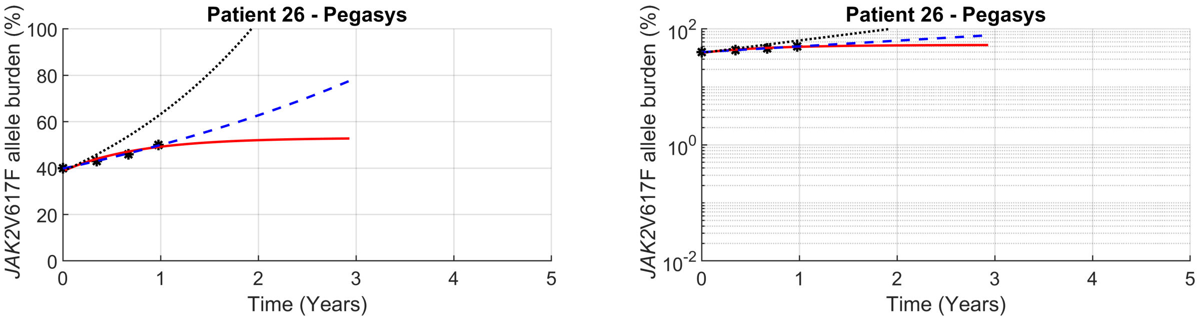

Supplemental figure 31: Data and model fits for patient 26, see legend of Supplemental figure 6

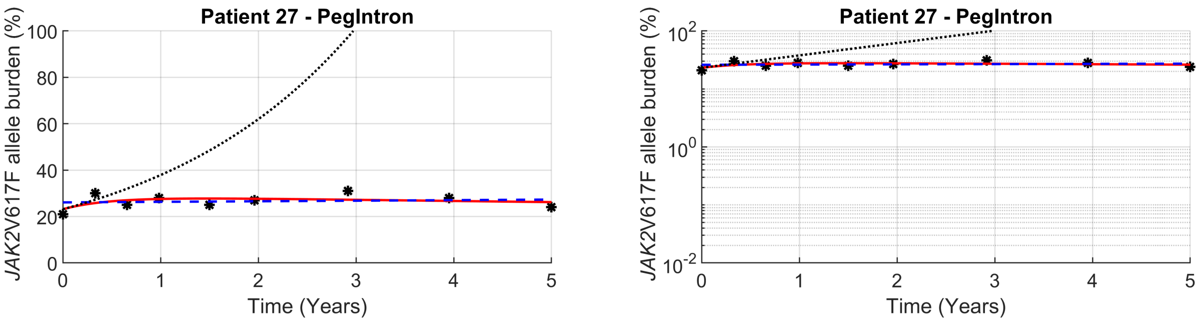

Supplemental figure 32: Data and model fits for patient 27, see legend of Supplemental figure 6

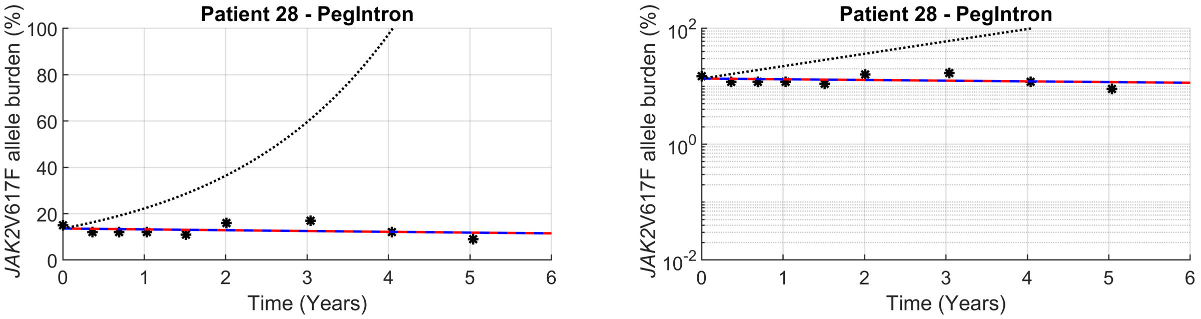

Supplemental figure 33: Data and model fits for patient 28, see legend of Supplemental figure 6

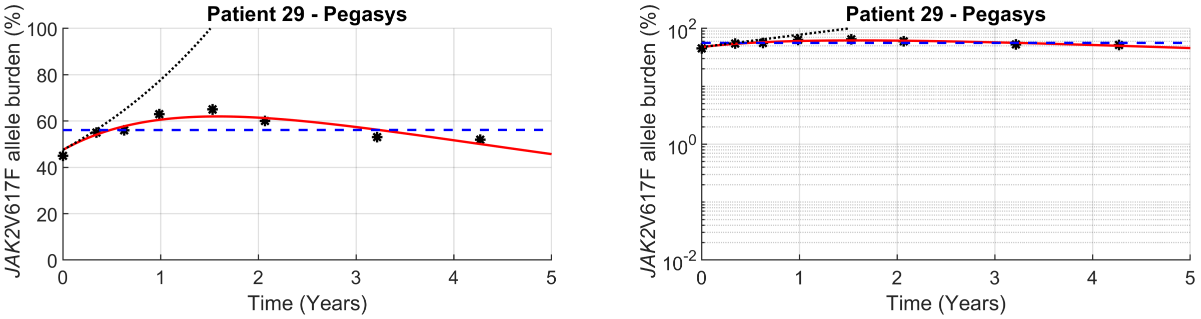

Supplemental figure 34: Data and model fits for patient 29, see legend of Supplemental figure 6

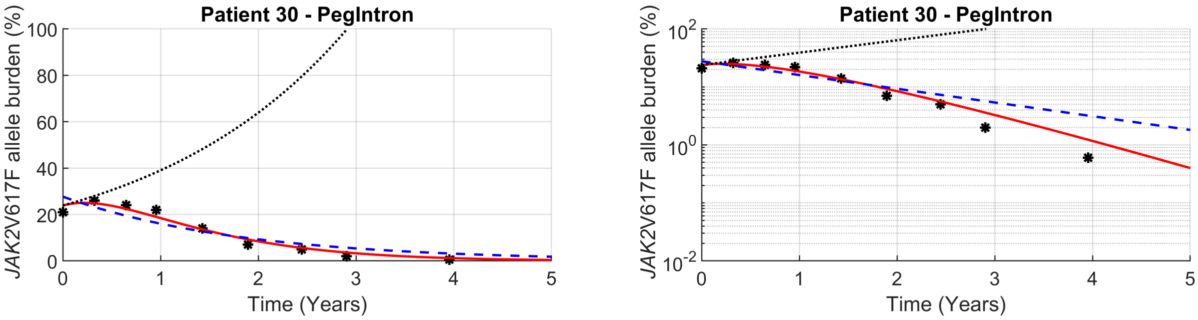

Supplemental figure 35: Data and model fits for patient 30, see legend of Supplemental figure 6

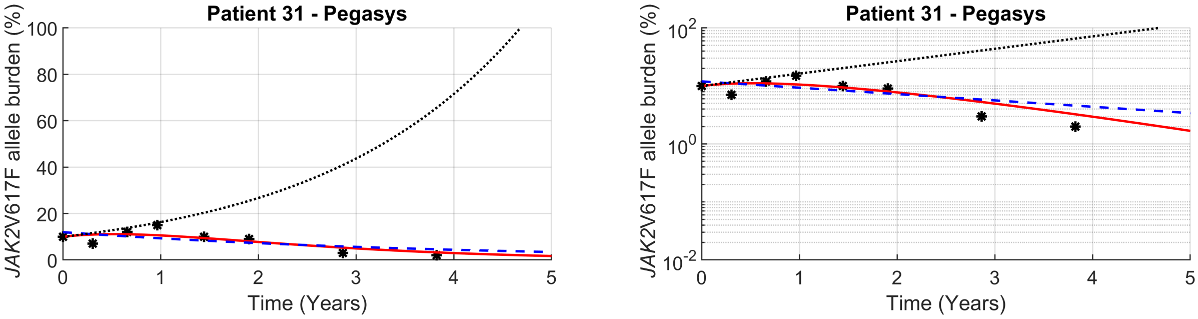

Supplemental figure 36: Data and model fits for patient 31, see legend of Supplemental figure 6

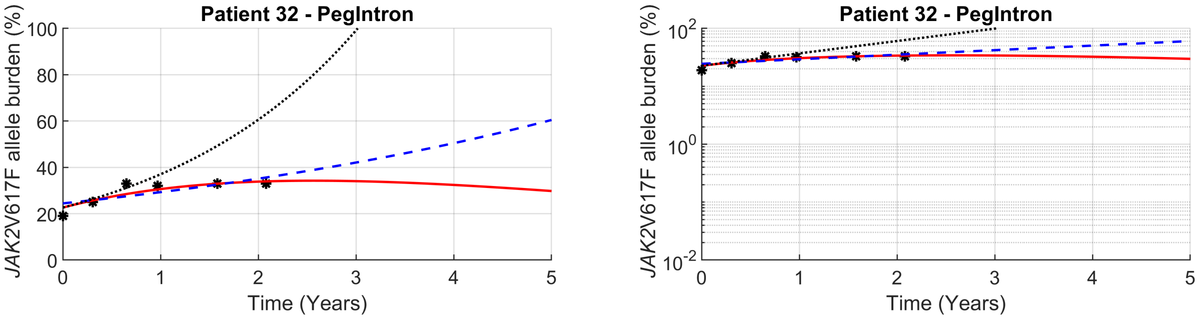

Supplemental figure 37: Data and model fits for patient 32, see legend of Supplemental figure 6

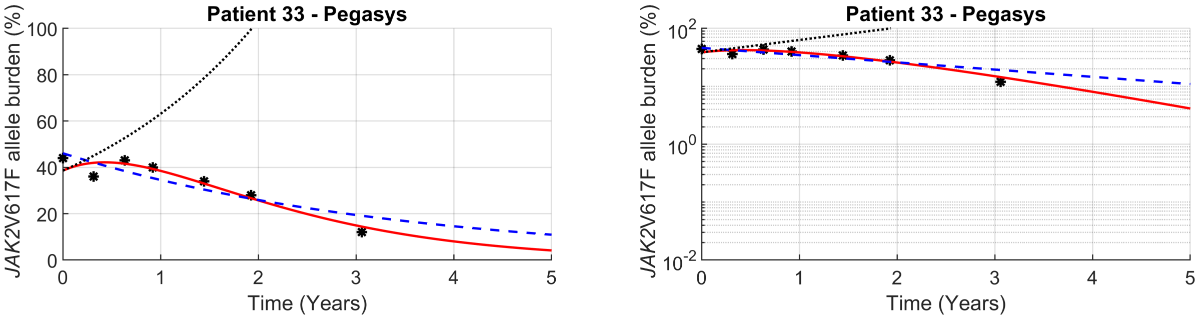

Supplemental figure 38: Data and model fits for patient 33, see legend of Supplemental figure 6

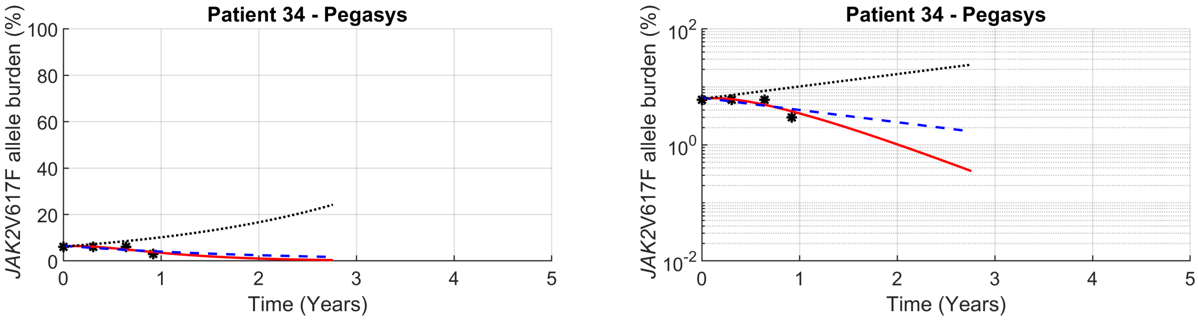

Supplemental figure 39: Data and model fits for patient 34, see legend of Supplemental figure 6

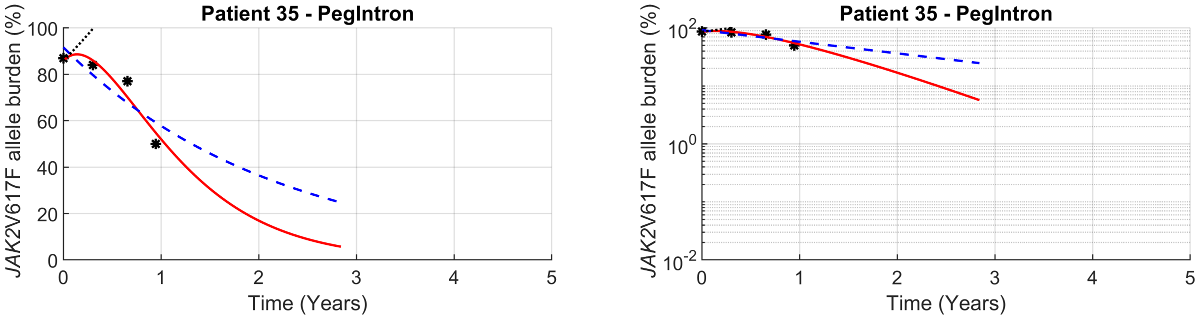

Supplemental figure 40: Data and model fits for patient 35, see legend of Supplemental figure 6

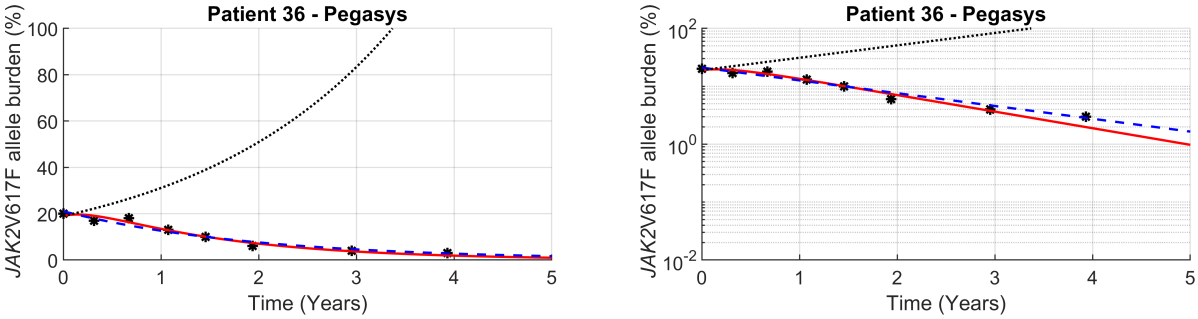

Supplemental figure 41: Data and model fits for patient 36, see legend of Supplemental figure 6

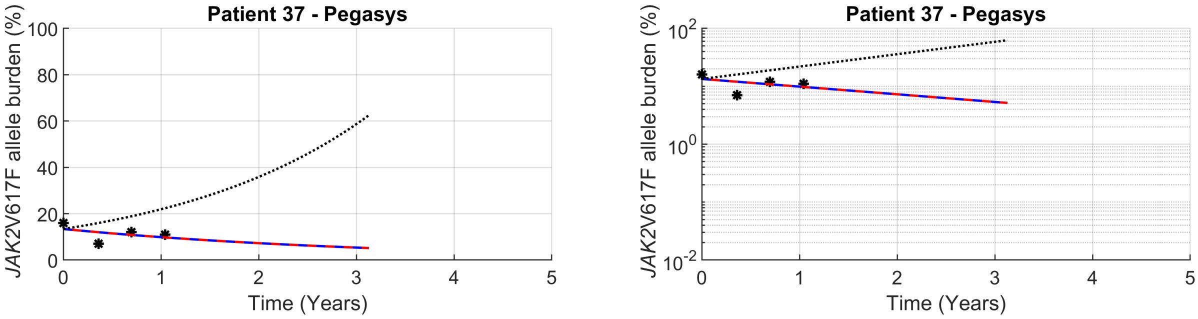

Supplemental figure 42: Data and model fits for patient 37, see legend of Supplemental figure 6

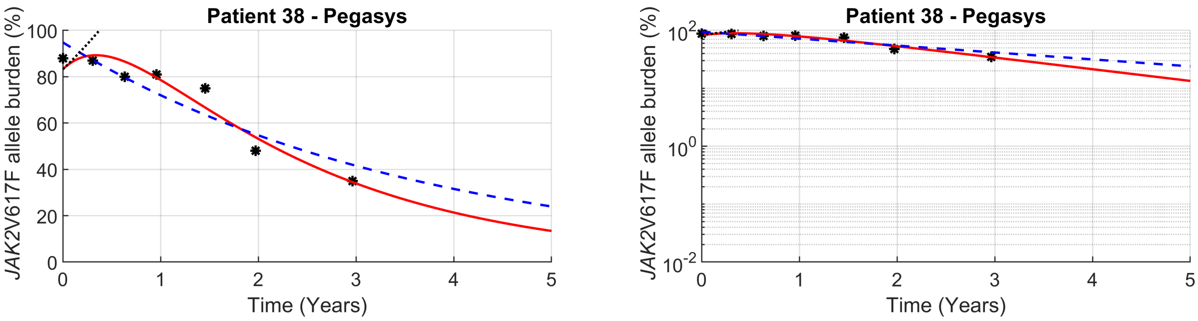

Supplemental figure 43: Data and model fits for patient 38, see legend of Supplemental figure 6

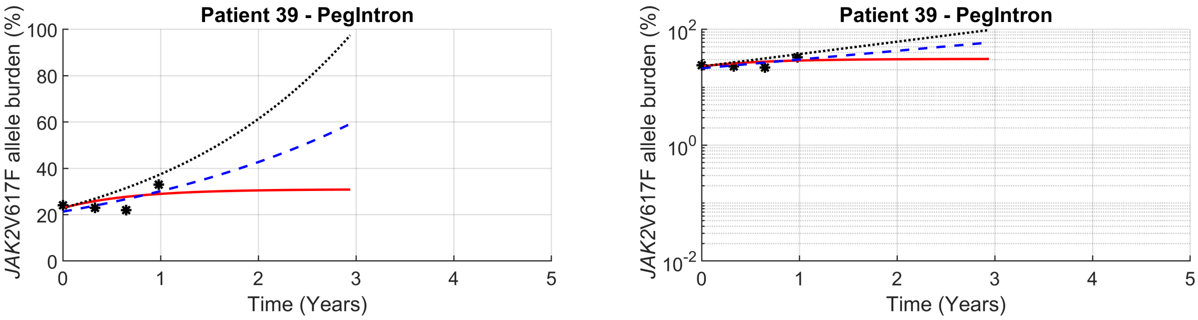

Supplemental figure 44: Data and model fits for patient 39, see legend of Supplemental figure 6

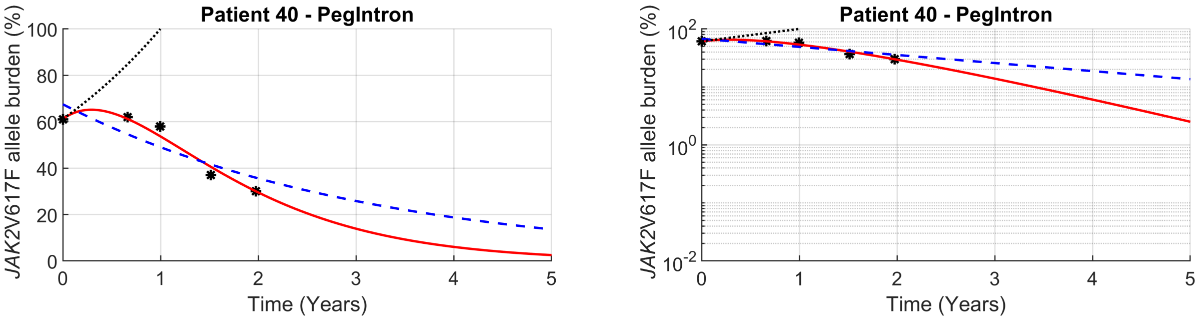

Supplemental figure 45: Data and model fits for patient 40, see legend of Supplemental figure 6

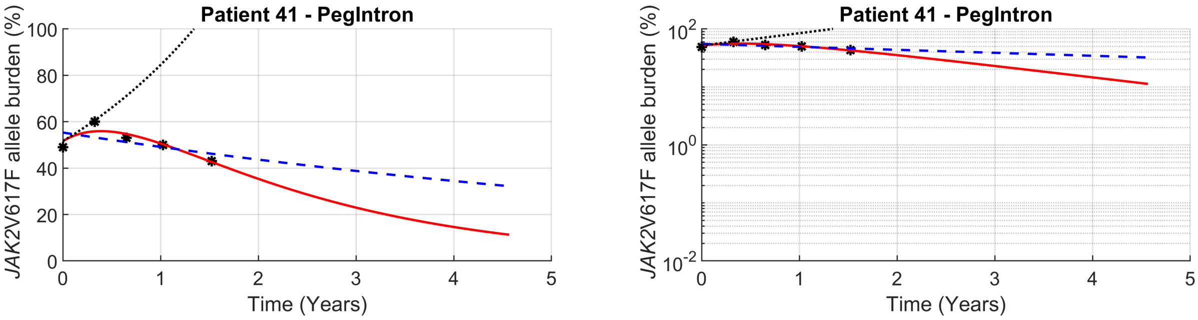

Supplemental figure 46: Data and model fits for patient 41, see legend of Supplemental figure 6

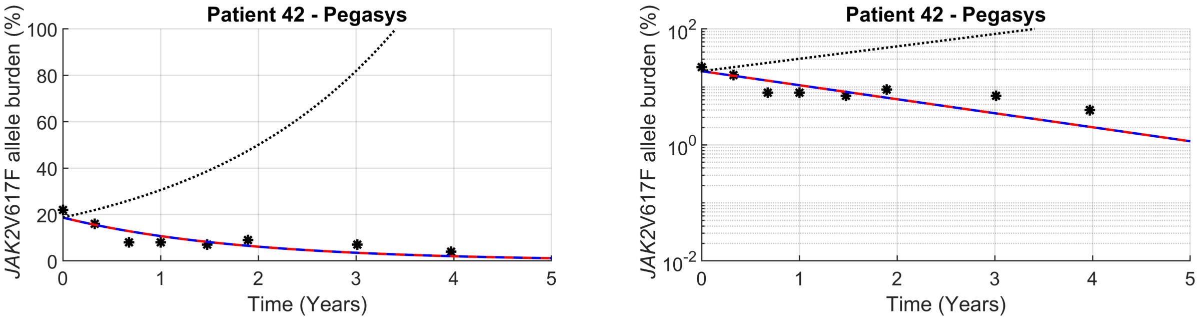

Supplemental figure 47: Data and model fits for patient 42, see legend of Supplemental figure 6

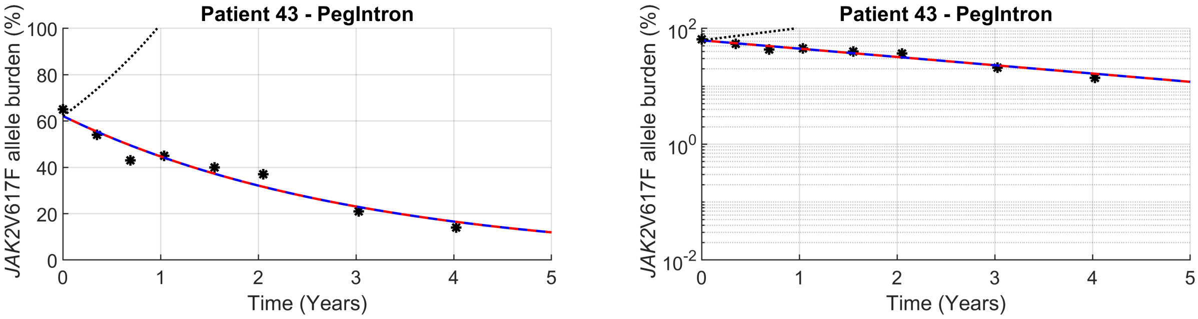

Supplemental figure 48: Data and model fits for patient 43, see legend of Supplemental figure 6

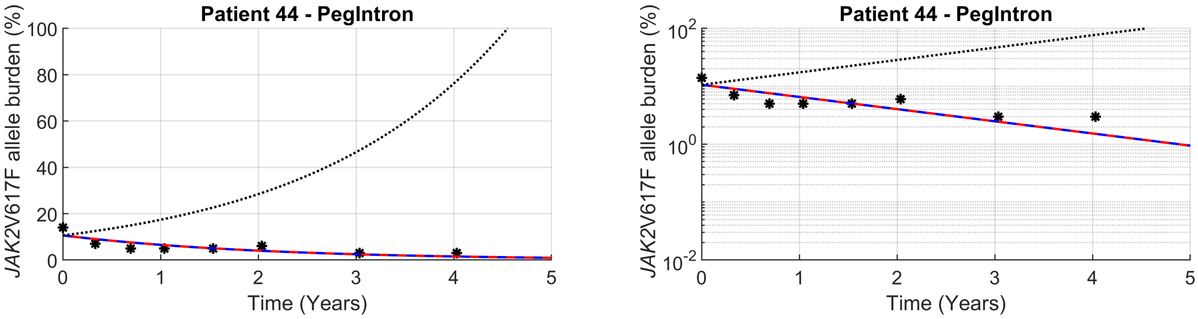

Supplemental figure 49: Data and model fits for patient 44, see legend of Supplemental figure 6

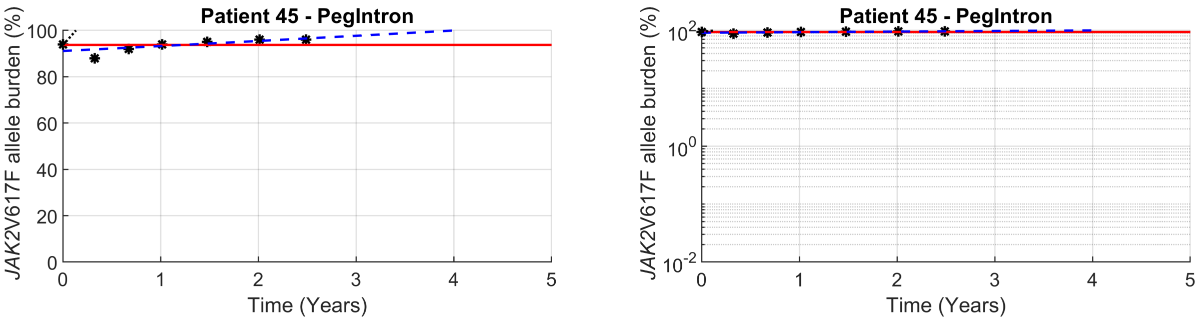

Supplemental figure 50: Data and model fits for patient 45, see legend of Supplemental figure 6

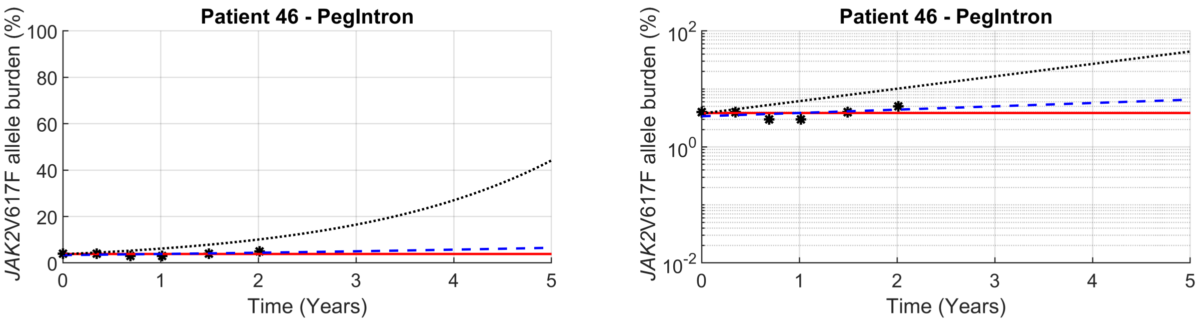

Supplemental figure 51: Data and model fits for patient 46, see legend of Supplemental figure 6

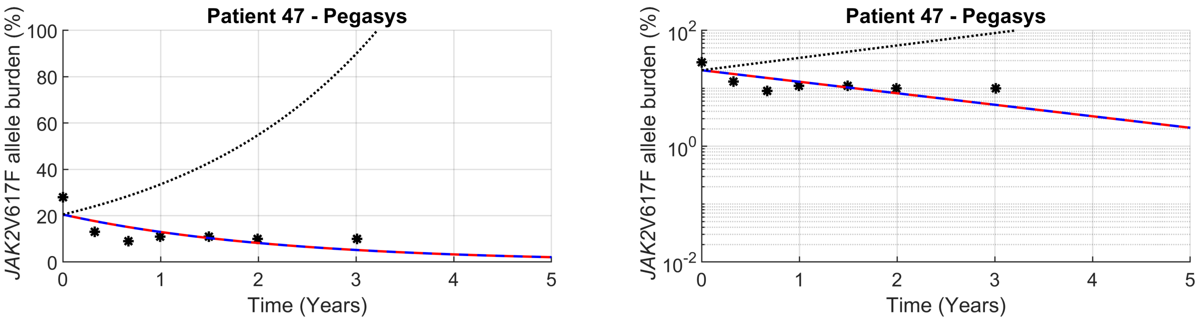

Supplemental figure 52: Data and model fits for patient 47, see legend of Supplemental figure 6

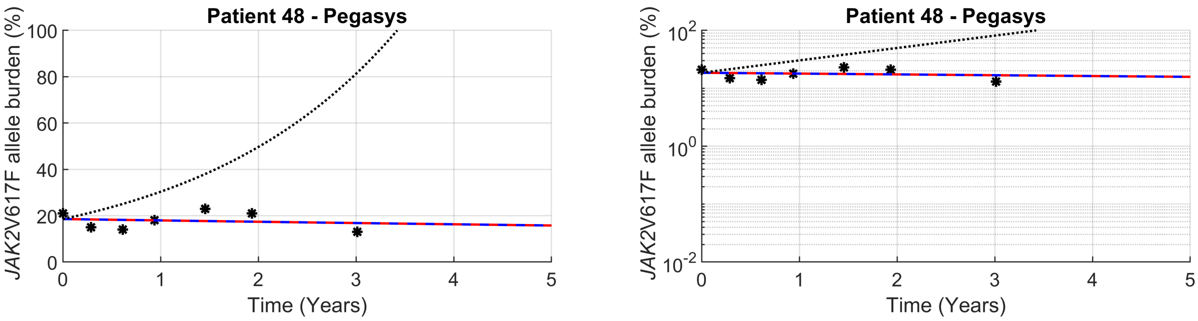

Supplemental figure 53: Data and model fits for patient 48, see legend of Supplemental figure 6

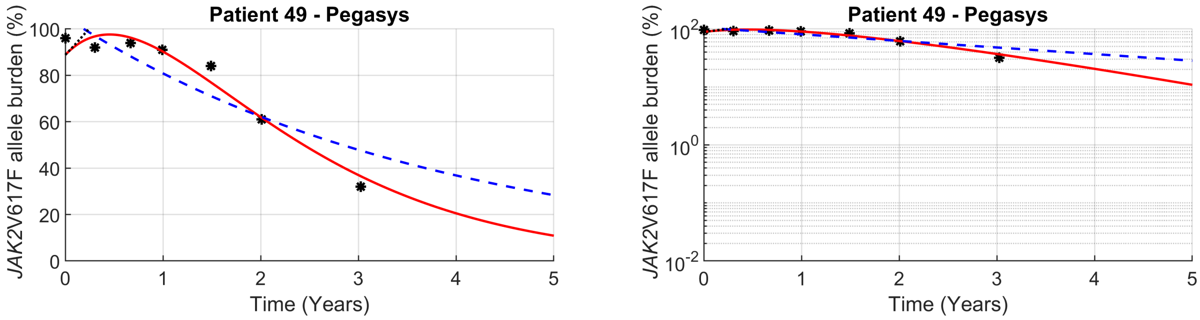

Supplemental figure 54: Data and model fits for patient 49, see legend of Supplemental figure 6

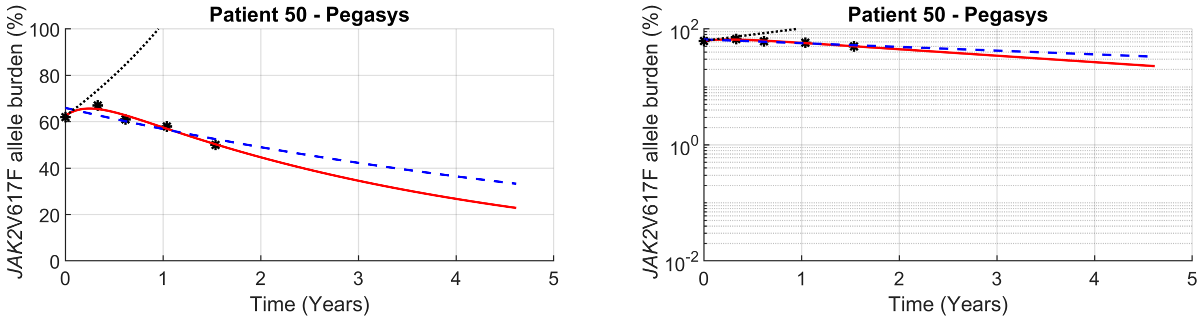

Supplemental figure 55: Data and model fits for patient 50, see legend of Supplemental figure 6

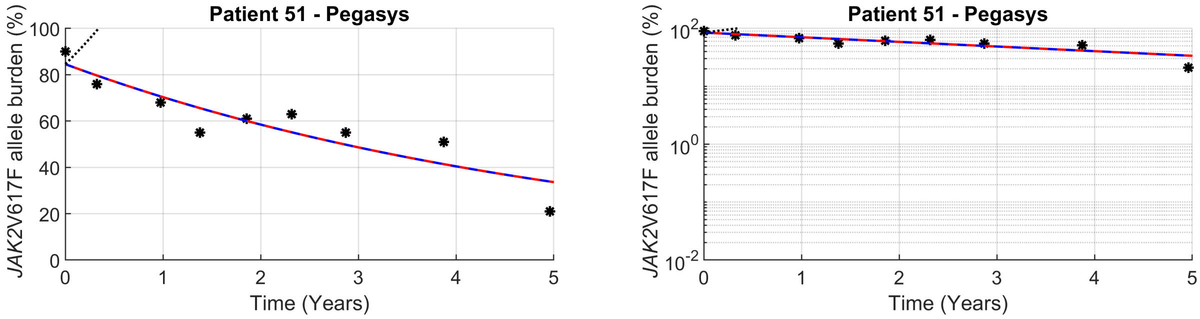

Supplemental figure 56: Data and model fits for patient 51, see legend of Supplemental figure 6

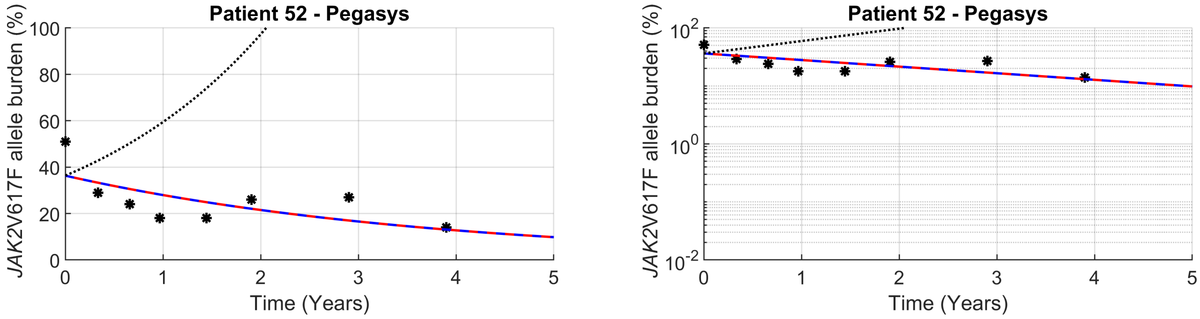

Supplemental figure 57: Data and model fits for patient 52, see legend of Supplemental figure 6

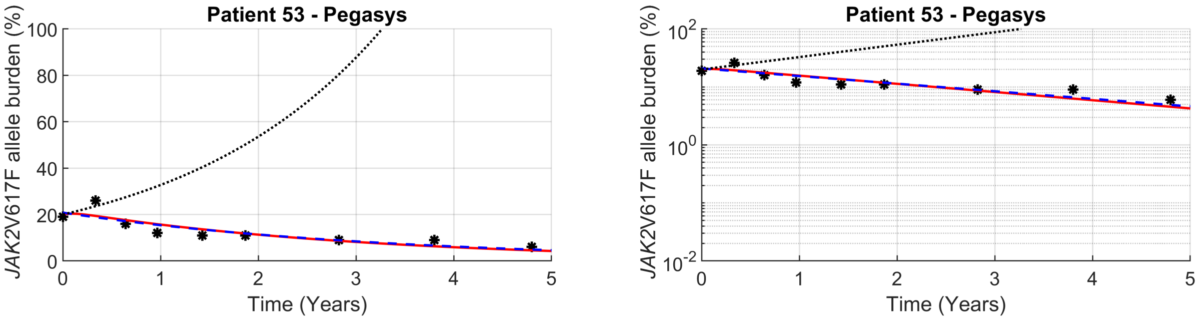

Supplemental figure 58: Data and model fits for patient 53, see legend of Supplemental figure 6

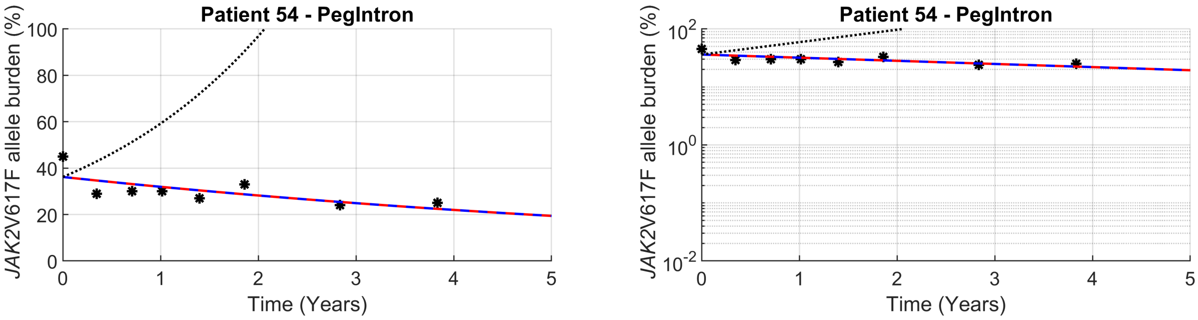

Supplemental figure 59: Data and model fits for patient 54, see legend of Supplemental figure 6

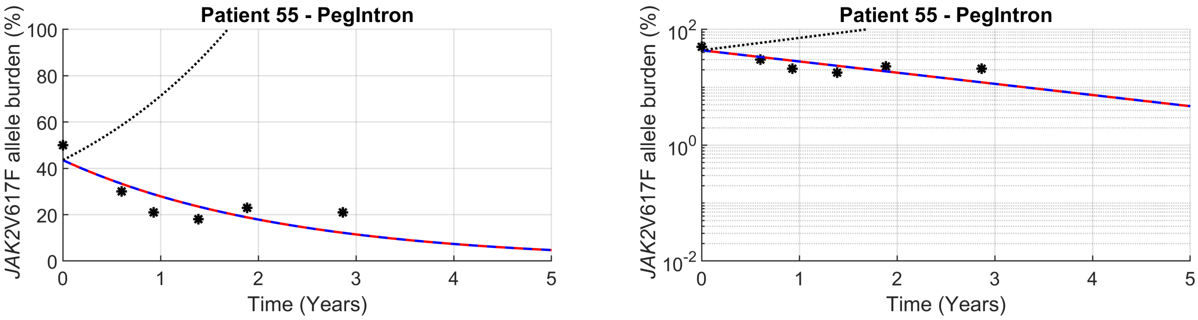

Supplemental figure 60: Data and model fits for patient 55, see legend of Supplemental figure 6

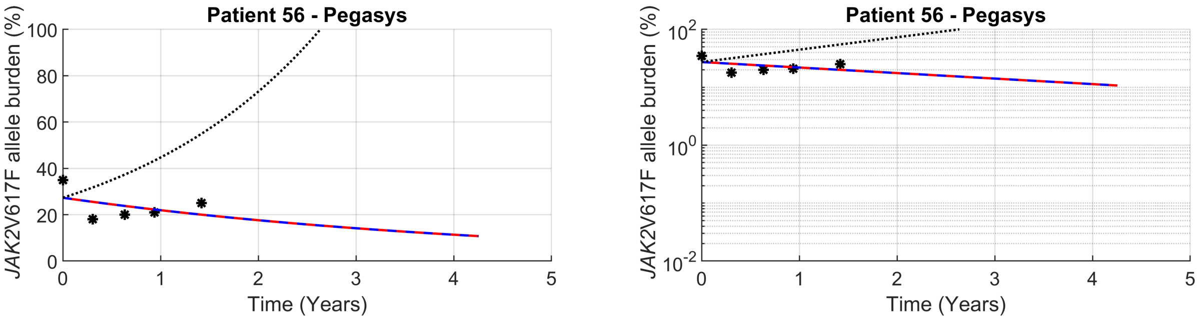

Supplemental figure 61: Data and model fits for patient 56, see legend of Supplemental figure 6

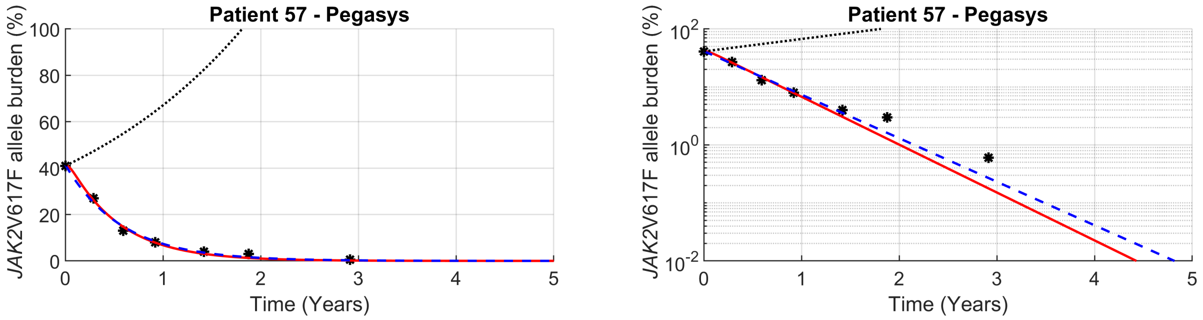

Supplemental figure 62: Data and model fits for patient 57, see legend of Supplemental figure 6

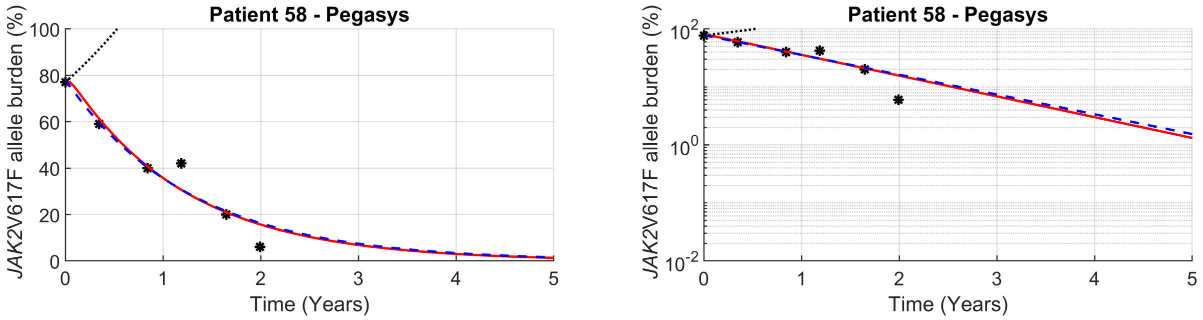

Supplemental figure 63: Data and model fits for patient 58, see legend of Supplemental figure 6

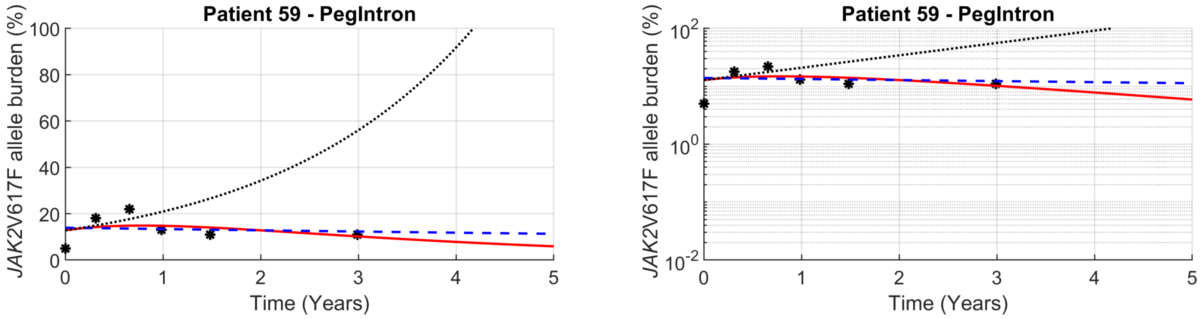

Supplemental figure 64: Data and model fits for patient 59, see legend of Supplemental figure 6

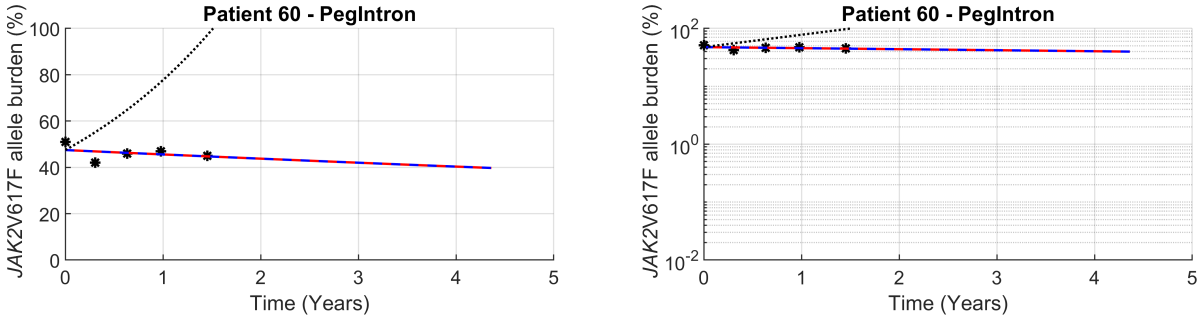

Supplemental figure 65: Data and model fits for patient 60, see legend of Supplemental figure 6

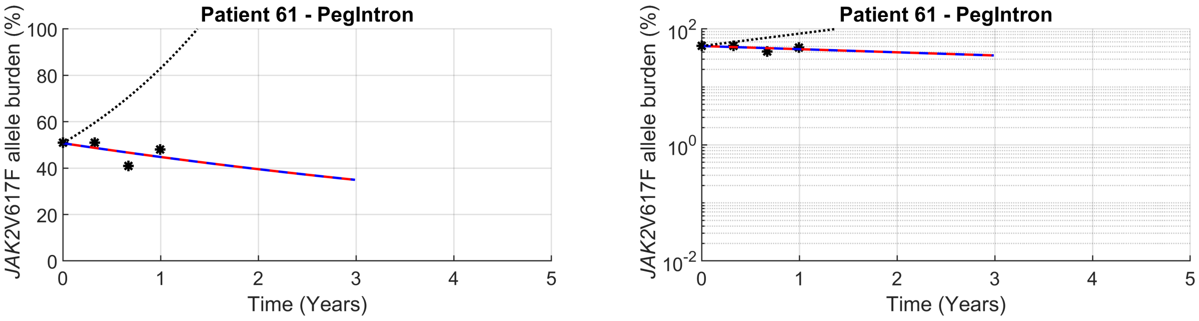

Supplemental figure 66: Data and model fits for patient 61, see legend of Supplemental figure 6

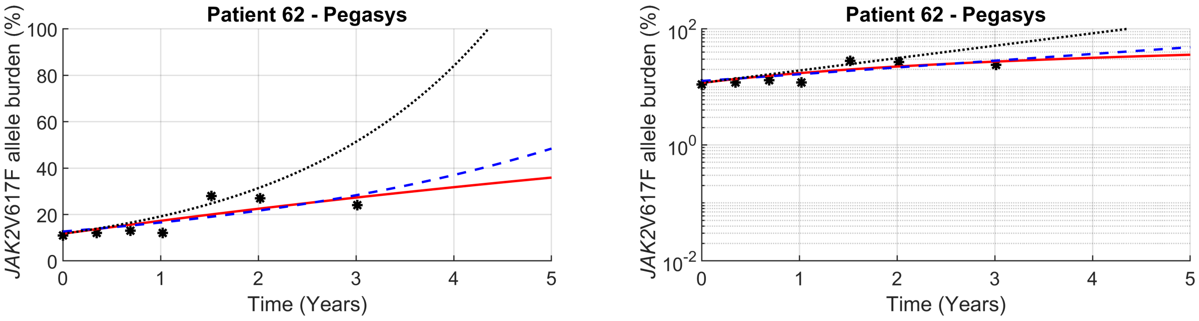

Supplemental figure 67: Data and model fits for patient 62, see legend of Supplemental figure 6

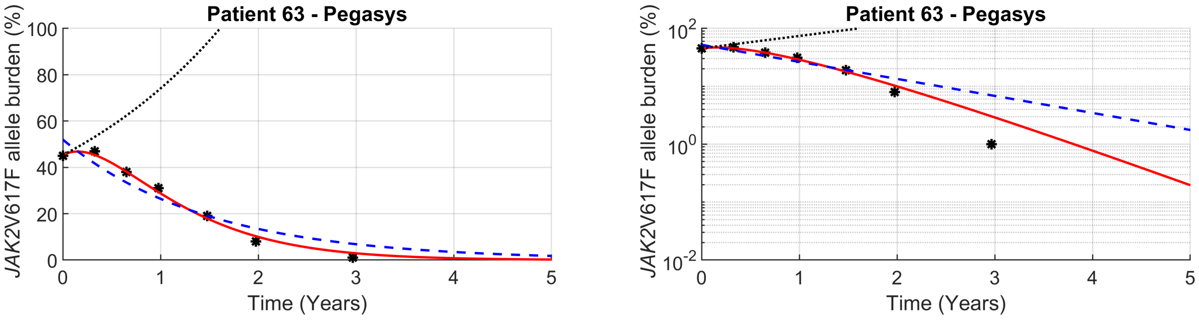

Supplemental figure 68: Data and model fits for patient 63, see legend of Supplemental figure 6

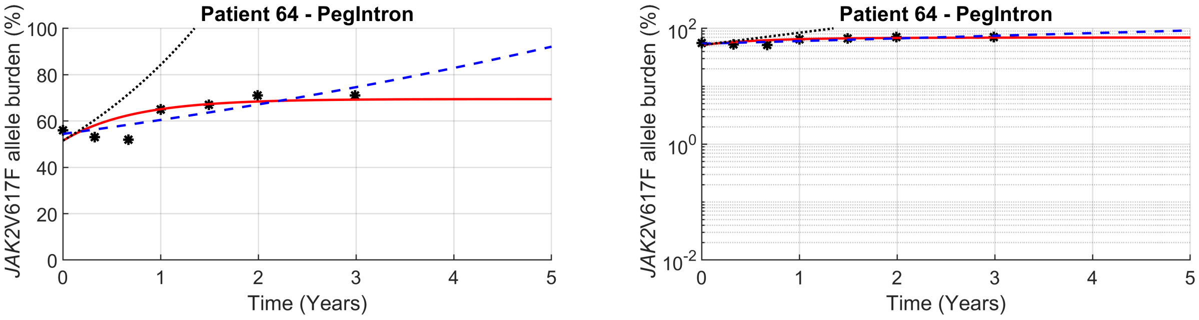

Supplemental figure 69: Data and model fits for patient 64, see legend of Supplemental figure 6

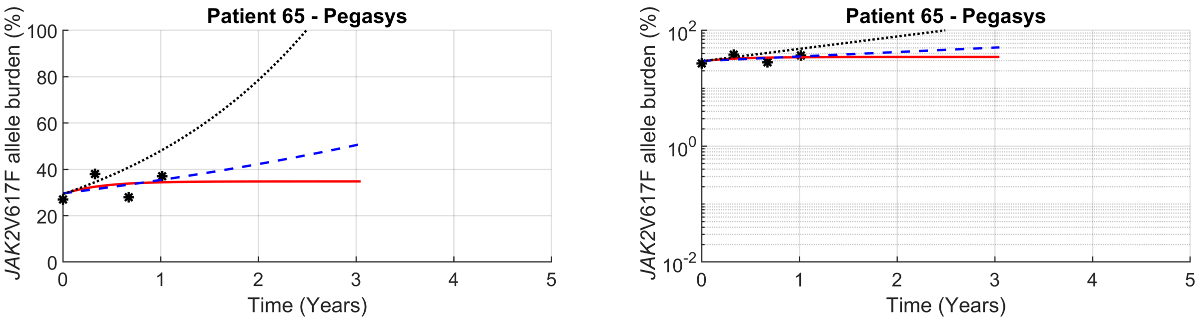

Supplemental figure 70: Data and model fits for patient 65, see legend of Supplemental figure 6

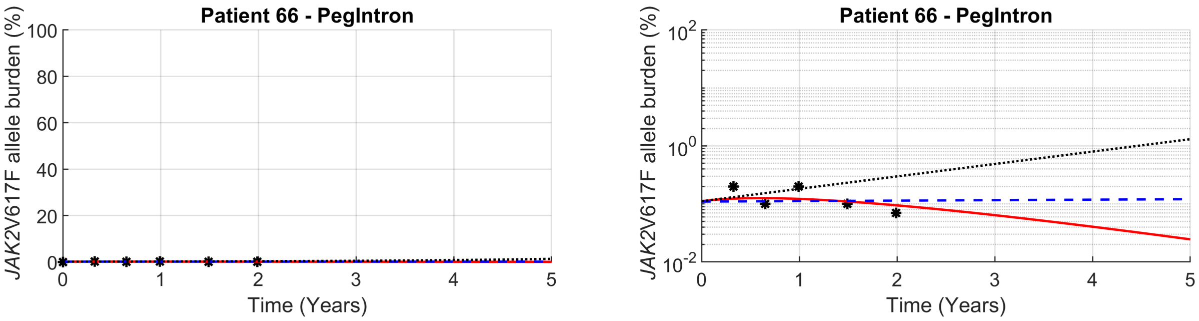

Supplemental figure 71: Data and model fits for patient 66, see legend of Supplemental figure 6

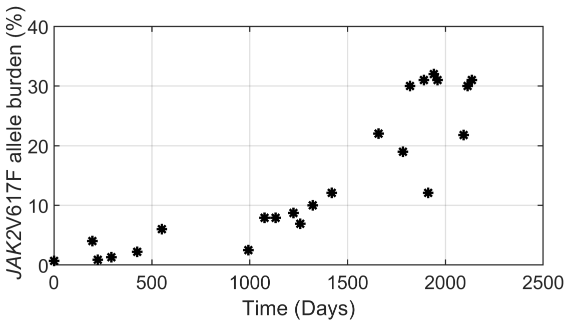

Supplemental figure 72: Raw data for patient A of the retrospective study.

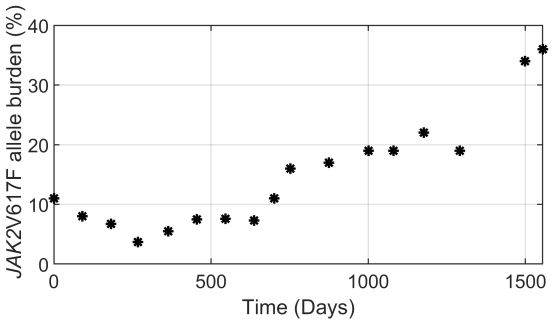

Supplemental figure 73: Raw data for patient B of the retrospective study.

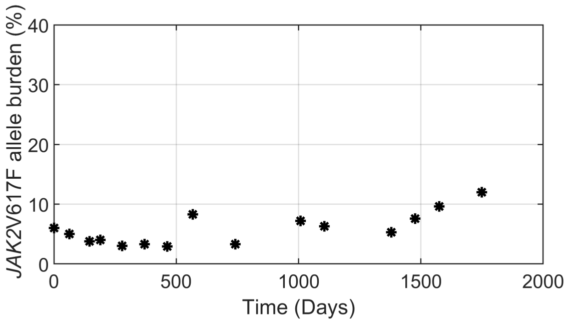

Supplemental figure 74: Raw data for patient C of the retrospective study.

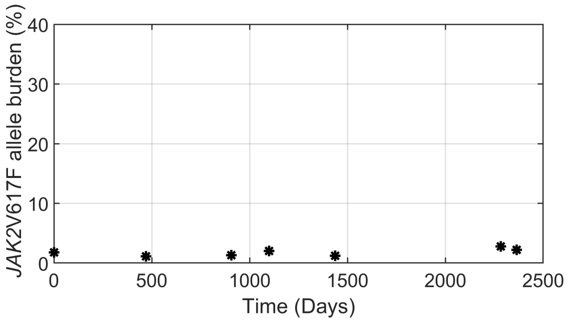

Supplemental figure 75: Raw data for the first of the two patients used as control for comparison only in the retrospective study.

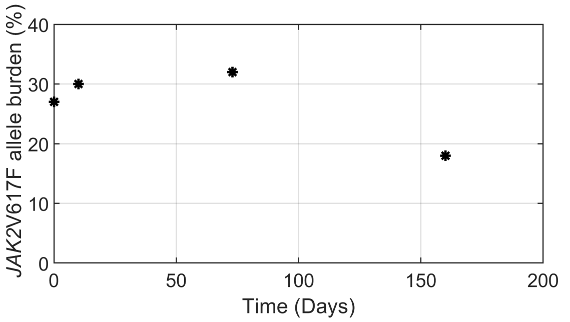

Supplemental figure 76: Raw data for the second of the two patients used as control for comparison only in the retrospective study.
